# Restitution properties during ventricular fibrillation and the effects of cardiac pacing location on ventricular electrophysiology in the rabbit heart

**DOI:** 10.1113/EP093784

**Published:** 2026-11-02

**Authors:** Bethan Roper‐Jones, Emily Allen, Rachel L. Sutcliffe, G. André Ng, Reshma A. Chauhan

**Affiliations:** ^1^ Department of Cardiovascular Sciences University of Leicester Leicester UK; ^2^ Leicester British Heart Foundation Centre of Research Excellence Leicester UK; ^3^ Leicester National Institute for Health and Care Research Biomedical Research Centre Leicester UK

**Keywords:** action potential restitution, and ventricular fibrillation, Cardiac electrophysiology, cardiac memory

## Abstract

Sudden cardiac death is a cause of mortality due to malignant arrhythmias such as ventricular fibrillation (VF). Although action potential duration (APD) restitution is a method to assess arrhythmic susceptibility, repolarisation may depend on prior activation history, a phenomenon known as cardiac memory. Cardiac memory describes the persistence of repolarisation effects from previous beats, such that APD reflects not only the immediately preceding diastolic interval (DI) but also earlier activation effects. This may be relevant during VF, where continuously varying activation patterns prevent a steady electrophysiological state. This study investigated how pacing location affects ventricular electrophysiology and whether repolarisation during VF exhibits short‐term cardiac memory. Langendorff‐perfused rabbit hearts were used to record monophasic action potentials from the left ventricular apex and base. Pacing was conducted from five sites. Steady‐state APD was assessed during constant pacing. Effective refractory period (ERP), APD restitution and conduction time restitution (CTR) were assessed using an S_1_–S_2_ extra‐stimulus protocol (S_1_, conditioning stimuli; S_2_, premature extra‐stimulus). Additionally, APD–DI and APD–APD relationships were analysed during early VF. Pacing location significantly influenced S_1_ and S_2_ conduction delays, consistent with site‐dependent activation timing. However, steady‐state APD, ERP and CTR were not significantly affected by pacing location. In contrast, during VF, APD exhibited a significant dependence on preceding DI and preceding APD over approximately the first 30 beats, with these relationships progressively deteriorating thereafter. These findings demonstrate that while pacing location alters activation timing, repolarisation dynamics under stable activation histories are robust. During VF, continuously changing activation patterns unmask short‐term cardiac memory.

## Abstract (highlights)

- **What is the central question of this study?**
  Does ventricular pacing location affect repolarisation under stable conditions, and does repolarisation during ventricular fibrillation depend on recent activation history?
- **What is the main finding and its importance?**
  Pacing location significantly altered conduction timing but did not affect steady‐state action potential duration (APD), restitution, effective refractory period or conduction time restitution (CTR). During early ventricular fibrillation (VF), APD showed a transient dependence on preceding diastolic interval and APD that diminished with time. This suggests that repolarisation is largely stable during paced rhythms, while short‐term history dependence becomes evident only during the early phase of VF.

## 1 INTRODUCTION

Sudden cardiac death (SCD) causes 90,000 deaths annually in the UK and remains an unresolved clinical issue (Ng, 2016). Malignant arrhythmias, particularly ventricular fibrillation (VF), are a leading cause of SCD (Hayashi et al., 2015). The mechanisms behind these arrhythmias are not fully understood, although increased electrical heterogeneity within the myocardium is thought to increase VF susceptibility. Current treatments are mostly palliative, including antiarrhythmic drugs and implantable cardioverter defibrillators (ICDs) (Fastenrath et al., 2026). ICDs, which deliver electrical shocks to correct VF or ventricular tachycardia (VT), have been shown to reduce mortality compared to drugs, especially in high‐risk or post‐sudden cardiac event patients. However, ICDs are expensive and not accessible to all patients, highlighting the need for improved risk stratification, which requires further understanding of arrhythmia (Ammannaya, 2020).

An important mechanism believed to influence arrhythmic susceptibility is the action potential duration restitution (APDR) properties of the heart. The heart's APDR property describes the relationship between action potential duration (APD) and the preceding diastolic interval (DI) (Taggart & Lab, 2008). Heart rate and premature stimuli influence APDR, with faster heart rates or premature stimuli shortening APD (Eisner et al., 2009). Although APD modulates refractoriness and excitation dynamics, heart rate in vivo is primarily determined by sinoatrial node activity; decreases in heart rate increase DI, which in turn leads to longer APD (Boyett et al., 2000; Mangoni & Nargeot, 2008). Since APD is directly related to preceding DI, a faster heart rate shortens DI, which in turn leads to a shorter APD (Wu & Patwardhan, 2004). This relationship forms a restitution curve, which can predict arrhythmogenesis through maximal slope analysis (Ng, 2016; Nolasco & Dahlen, 1968). A steeper slope indicates a greater change in APD with small DI changes, increasing the likelihood of wavebreak, which can lead to ventricular fibrillation (VF) (Qu et al., 1999). However, restitution measurements are protocol‐dependent and may not fully capture electrophysiological behaviour during complex rhythms such as VF (Iravanian et al., 2023).

In addition to restitution effects, repolarization may depend on prior activation history, a phenomenon commonly referred to as cardiac memory (Rosen & Bergfeldt, 2015). Importantly, cardiac memory encompasses at least two distinct processes. The first is activation sequence‐dependent memory, which develops over prolonged periods of altered activation and is associated with ion channel remodelling, including changes in currents such as transient outward potassium current (*I*_to_) and slow delayed rectifier potassium currents (*I*_Ks_). The second is short‐term, beat‐to‐beat memory, in which APD depends not only on the immediately preceding DI but also on earlier activation events (Huang et al., 2004; Rosen, 2000; Rosen & Bergfeldt, 2015). The exact molecular mechanisms of cardiac memory are still not fully understood. Classical activation sequence‐dependent cardiac memory has been proposed to involve local mechanical myocardial stretch during altered activation, triggering intracellular signalling pathways that reduce repolarisation currents, including *I*_to_ in species where it is prominently expressed (Shvilkin et al., 2015). In contrast, the mechanisms underlying short‐term, beat‐to‐beat cardiac memory remain less well defined and are thought to reflect incomplete recovery of ionic currents and calcium handling between successive beats. Cardiac memory has been investigated during VF in a porcine model with an influence of preceding APD and DIs having an impact on subsequent APDs and DIs (Huang et al., 2004). As porcine ventricular myocardium has minimal expression of *I*_to_, these findings suggest that short‐term cardiac memory during VF is not solely dependent on *I*_to_‐mediated mechanisms. While cardiac memory has been demonstrated during pacing and following altered activation sequences, its role during VF remains incompletely defined. The present study focuses on this short‐term form of cardiac memory, particularly under the dynamic conditions of VF, where activation history varies continuously.

Pacing experiments impose a stable activation history characterised by fixed wavefront origin, predictable cycle length and consistent electrotonic loading (Osadchii, 2012; Ramlugun et al., 2023). In contrast, VF represents an extreme electrophysiological state in which activation origin, direction, timing and electrotonic interactions vary continuously from beat to beat, reflecting a transition to spatiotemporal chaos (Chattipakorn et al., 2005; Lee et al., 2001; Qu et al., 1999). Under such conditions, the myocardium is prevented from reaching a steady state, potentially unmasking history‐dependent repolarization dynamics that are not apparent during pacing (Eisner et al., 2009; Iravanian et al., 2023; Rosen & Bergfeldt, 2015).

Pacing the heart at a rate above its intrinsic rhythm can induce changes in APD. The pacing location itself can also influence AP morphology and duration, and these effects vary across cardiac regions due to the heterogeneous distribution of ion channels (Ahlberg et al., 2007, 2008; Choi & Salama, 2000; Elliott et al., 2022; Osadchii, 2012; Pitruzzello et al., 2007; Wood et al., 1996). The rabbit ventricle, as used in the present study, exhibits species‐specific ionic current expression that differs from the human heart. In particular, ventricular repolarisation in rabbits relies more heavily on *I*_Kr_ and *I*_Ks_, with comparatively reduced transmural heterogeneity and differences in the distribution of *I*_to_ relative to humans. These differences influence action potential morphology, restitution behaviour and repolarisation gradients, and should therefore be considered when interpreting the translational relevance of the present findings (Antzelevitch, 2000; Bartos et al., 2015; Szentadrassy et al., 2005). This regional heterogeneity can be associated with sudden cardiac death and arrhythmias (Prenner et al., 2016).

Most APDR studies use epicardial pacing, particularly from the left ventricle (LV), which is largely due to its experimental accessibility and ability to generate larger gradients of repolarisation (Elliott et al., 2022; Ng et al., 2001). Computational models suggest greater repolarization dispersion when pacing from the epicardium compared to the endocardium (Mendonca Costa et al., 2019; Pitruzzello et al., 2007), potentially exaggerating physiological effects and limiting clinical translatability, given that normal activation originates endocardially from the sinoatrial node.

Additional studies comparing left and right ventricular pacing have identified differences in arrhythmia susceptibility and apex–base heterogeneities. Specifically in humans, the apex has a larger *I*_to_, while the base shows a larger *I*_Ks_ (Bartos et al., 2015; Szentadrassy et al., 2005). These apex–base heterogeneities may further influence APD and pacing responses. Recent studies also highlight that pacing location impacts VF induction susceptibility, with differences found between the left and right ventricles (Ahlberg et al., 2008; Choi & Salama, 2000). Despite these complexities, few studies have investigated the impact of pacing location, specifically remote or endocardial pacing, on the modulation of electrophysiological properties, with the large number of existing studies using LV epicardial pacing (Elliott et al., 2022; Osadchii, 2012).

Although pacing location is known to influence activation sequence and conduction delay, its effect on restitution properties remains unclear, particularly when comparing endocardial and epicardial stimulation. Moreover, few studies have directly contrasted electrophysiological behaviour under stable pacing conditions with that observed during VF within the same experimental preparation. Therefore, this study had two primary aims. First, to investigate the effect of pacing location on ventricular electrophysiology under stable activation conditions, including restitution properties and conduction delay. Second, to examine APD–DI relationships during the early phase of VF to assess the presence of short‐term, beat‐to‐beat cardiac memory under dynamically varying activation conditions. Using a Langendorff‐perfused rabbit heart model, we combined controlled pacing and VF analysis to compare electrophysiological behaviour under stable and highly variable activation states.

## 2 METHODS

### 2.1 Ethical approval

Ethical approval was ensured prior to study completion conforming to the Animals (Scientific Procedures) Act 1986, the Animal Welfare Act 2006, and the *Guide for the Care and use of Laboratory Animals* from the US National Institute of Health (National Research Council, 2011). This study was completed following ethical approval from the University of Leicester animal welfare review board (AWERB) under the Home Office Project Licence PP4614103. The investigators of this study understand the ethical principles under which *Experimental Physiology* operates and the work of this study complies with the animal ethics checklist.

### 2.2 Langendorff whole heart isolation

Adult male New Zealand White rabbits (*n* = 66, 3–3.5 kg, Inotiv, West Lafayette, IN, USA) were kept with lighting on a 12/12‐h light/dark cycles, with ad libitum feeding, humidity 50% and room temperature 18°C. Rabbits were sedated via subcutaneous injection using a combination of medetomidine hydrochloride (Sedator, 0.2 mg/kg, Dechra, Shrewsbury, UK), butorphanol (Torbugesic, 0.05 mg/kg, Zoetis, Parsippany, NJ, USA) and ketamine (Ketavet, 10 mg/kg, Zoetis, Parsipanny, NJ, USA). Following successful sedation, the marginal ear vein was cannulated for the delivery of heparin (1000 IU, Multiparin, Wrexham, UK) and a lethal overdose of pentobarbital sodium (Dolethal, 160 mg/kg, Vetoquinol, Towcester, UK). The heart was isolated and placed into cold Tyrode solution (in mM: Na^+^ 138.0, K^+^ 4.0, Mg^2+^ 1.0, Ca^2+^1.8, H_2_PO_4_^−^ 0.4, Cl^−^ 121.0, HCO_3_^−^ 24.0, and C_6_H_12_O_6_ 11.0.) as previously published (Ng et al., 2001).

### 2.3 Langendorff perfusion

The heart was perfused via the ascending aorta with Tyrode solution (pH 7.4, 37°C), bubbled with carbogen (95% O_2_/5% CO_2_) at a flow rate of 40 mL/min using a Gilson MINIPULS® 3 peristaltic pump (Gilson Inc., Middleton, WI, USA). A drain was placed in the left ventricle apex for thebesian venous effluent drainage. Perfusion pressure was monitored with a pressure transducer (MTL0380, ADInstruments Ltd, Oxford, UK) connected in series to the aortic cannula. Left ventricular pressure was also measured through a similar pressure transducer connected to a latex balloon filled with distilled water, to give an end‐diastolic pressure between 0 and 5 mmHg.

### 2.4 Cardiac electrical pacing and recording

Hook electrodes and a bipolar catheter were used for pacing at double the diastolic pacing threshold through a constant current stimulator (DS7A, Digitimer, Welwyn Garden City, UK). The pacing catheter was placed endocardially (endo) at the right ventricle (RV) apex and hook electrodes were placed in the epicardial (epi) tissue of the RV apex, RV base, LV apex and LV base. The order of hook electrode placement was randomised. Constant pacing was at a fixed cycle length (CL) of 300 ms until APD stabilised (approximately 90 s). Two contact electrodes were used to record monophasic action potentials (MAP) from the epicardium of the LV, one apical and the other basal, utilising a DC‐coupled high input impedance differential amplifier (Biomedical Joint Workshop, University of Leicester, UK). A single apical and basal LV epicardial recording site was used to provide high‐fidelity local measurements of repolarisation and conduction while maintaining signal stability during prolonged VF recordings. This configuration was selected to ensure consistent signal quality across all pacing conditions and to minimise spatial variability introduced by multi‐site mapping during unstable rhythms such as VF. Pacing sites were selected to provide distinct ventricular activation pathways and varying activation distances between the pacing and recording locations, allowing the influence of activation sequence on conduction and repolarisation to be assessed relative to the LV epicardial recording sites.

### 2.5 Action potential duration restitution

Electrical restitution data were collected through an extra stimulus pacing protocol from multiple pacing sites. Hearts were paced for 20 beats (S_1_) at a CL of 300 ms, then an extra stimulus (S_2_) was delivered. The S_2_ CL was initially 300 ms and then progressively decreased: initially by 10 ms until 200 ms, then by 5 ms until the ventricular effective refractory period (ERP), which is the failure to capture the S_2_ stimuli. MAP duration was analysed, using a custom‐written program (Francis Burton, Glasgow University, UK) (Kettlewell et al., 2004). MAP duration was measured from the initiation of a signal to 90% repolarisation (MAPD_90_) as previously described (Chin et al., 2023). Due to the absence of a fixed cycle length during ventricular fibrillation, classical restitution curves could not be constructed. Instead, APD–DI relationships were analysed to assess restitution‐like dynamics under these conditions. An exponential curve was determined using the equation: MAPD_90_ = MAPD_90max_(1 − e^−DI/τ^), with τ being the time constant and fitted with Origin (v10.0, OriginLab Corp., Northampton, MA, USA). A derivative curve was then produced and the maximum slope of restitution determined and analysed. Conduction time restitution (CTR) analysis was assessed using S_2_ delay across coupling intervals during the ERP protocol. An exponential curve was generated from the equation: S_2__delay_ = S_2__delaymax_(1 − e^−S1–S2interval/τ^). Derivative curves were also produced and analysed. Because only single‐site monophasic action potential recordings were available, conduction time was assessed from the interval between the pacing stimulus and the local MAP upstroke. Although this approach does not provide true conduction velocity or absolute conduction time over a known distance, it provides a robust surrogate measure of local activation timing, allowing within‐preparation comparisons of relative conduction changes across pacing sites and coupling intervals. All curves were produced using Origin.

### 2.6 Ventricular fibrillation threshold

A train of 20 stimuli was delivered at 300 ms CL, which was then followed by a 30 stimuli train at a CL of 30 ms via endocardial pacing at the RV apex. The pacing train begins at a current of 0.5 mA and then increases by 0.5 mA following each 50‐beat train and 5 ms rest period until sustained VF. The current required to initiate VF is referred to as the VF threshold (VFT). VF was sustained for 2 min.

### 2.7 Data recording and analysis

All signals were recorded through a PowerLab 800/s system (ADInstruments) and digitised at 2 kHz through LabChart software (v8.0, ADInstruments). S_1_ and S_2_ delay were calculated through a custom‐written program and displayed as the mean ± standard deviation. Statistical analysis was performed using one‐way ANOVA (GraphPad Prism v.10.0, GraphPad Software, Boston, MA, USA). Six hearts were studied in total.

VF analysis was specifically used to assess short‐term, beat‐to‐beat cardiac memory, whereas pacing protocols were used to evaluate steady‐state electrophysiological properties under fixed activation conditions. VF data were analysed with APD_50_. The APD_50_ was determined from the last normal AP prior to VF initiation (Appendix Figure A1). *T*_act50_ (the crossing point during depolarisation) and *T*_50_ (the crossing point during repolarisation) were determined by the time point at which the following AP crossed the predetermined APD_50_ through MATLAB T2023b software (The MathWorks Inc., Natick, MA, USA). All *T*_act50_ and *T*_50_ points were also manually checked through LabChart software (ADInstruments). The *T*_act50_ and *T*_50_ were then used to determine the APD and DI of the VF action potentials. Short low‐amplitude MAPs observed during VF were omitted from the analysis as they did not meet the APD_50_ threshold. For classification, the reference APD_50_ was denoted APD_50(_ *_x_*_)_ for a given action potential, with preceding APs labelled APD_50(_ *_x_*_−1)_ through to APD_50(_ *_x_*_−5)_) representing the first to the fifth immediately preceding action potentials. Corresponding DI values (DI_(_ *_n_*_−1)_ to DI_(_ *_n_*_−5)_) were defined as the interval between the APD_50_ of a preceding beat and the subsequent upstroke up to the fifth immediately preceding (Figure A1). APD_50_ values exceeding 500 ms were considered non‐physiological and excluded from analysis as they likely represent signal noise or MAP detection artefacts during low‐amplitude VF signals.

## 3 RESULTS

### 3.1 The effect of constant pacing from different locations on APD

The mean APD values during constant pacing recorded at the MAP apex are shown for different pacing sites (in parentheses):131.6 ± 14.7 ms (RV apex endo), 123.4 ± 17.8 ms (RV apex epi), 125.3 ± 9.8 ms (RV base epi), 127.3 ± 17.5 ms (LV base epi) and130.3 ± 9.8 ms (LV apex epi) as illustrated in Figure 1b,c (exact *P*‐values shown in Table 1) and Appendix Figure A2. The mean APD values during constant pacing when recording at the MAP base were 123.2 ± 12.6 ms (RV apex endo), 123.8 ± 8.3 ms (RV apex epi), 136.3 ± 7.9 ms (RV base epi), 128.8 ± 14.5 ms (LV base epi) and 136.2 ± 10.7 ms (LV apex epi). Pacing at different locations resulted in no significant difference in APD at apex or base. Both before pacing and during pacing APD was analysed but no significant differences were identified between the five pacing locations for either apex or base recording locations (all *P* ≥ 0.093) (Table 2).

**FIGURE 1:**
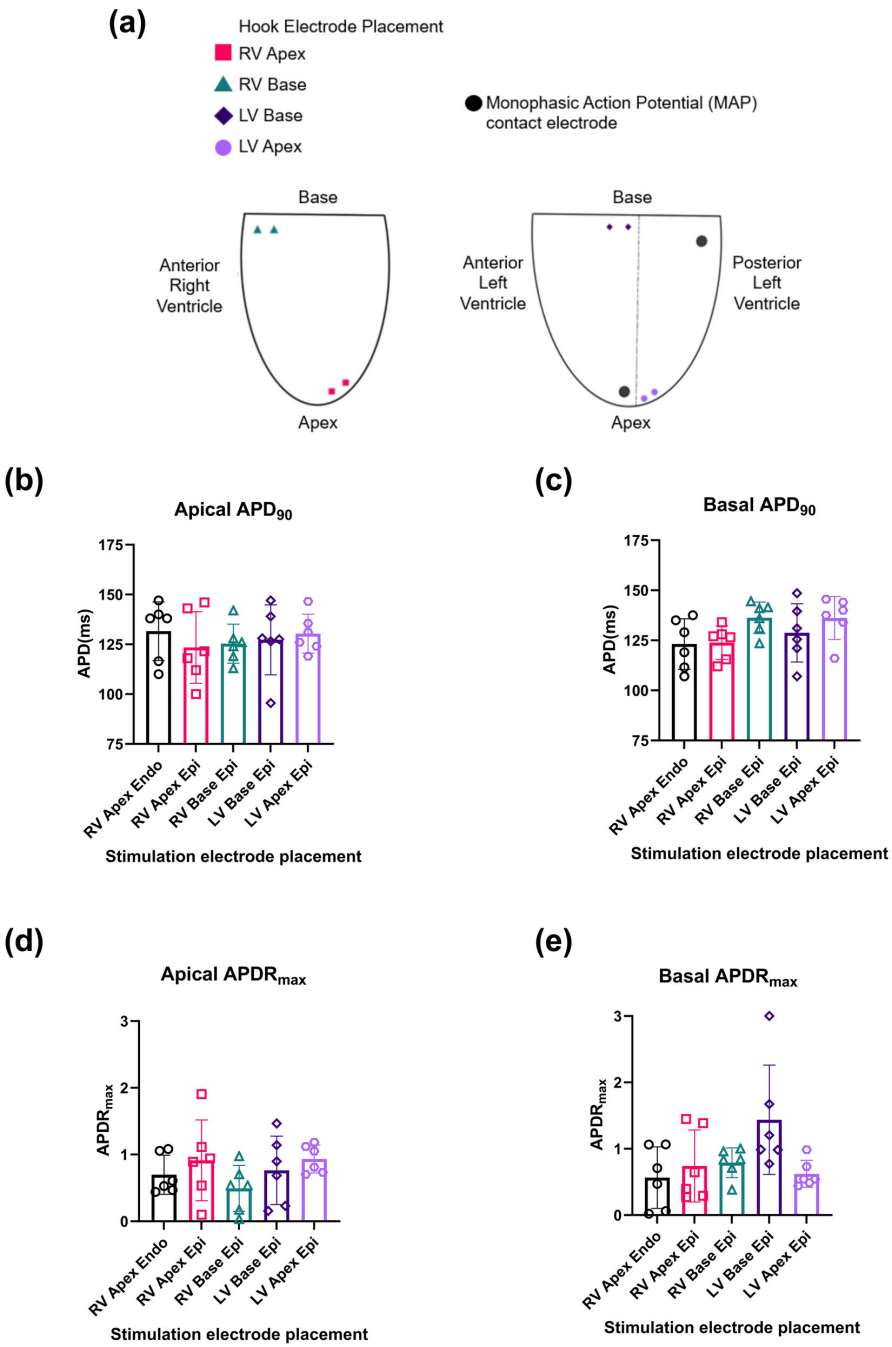
Effect of stimulation electrode placement on APD_90_ (MAPD_90_) and APDR_max_. (a) An illustration of the hook electrode placement for stimulation and monophasic action potential (MAP) contact electrode recording locations on the anterior and posterior left and right ventricle. (b, c) The APD_90_ during constant pacing at 300 ms for all five stimulation locations from the apical (b) and basal (c) recording MAP contact electrodes. (d, e) The action potential duration restitution maximum (APDR_max_) from the apical (d) and basal (e) MAP contact electrode. Individual values are plotted for each electrode placement with bars representing means ± SD (*n* = 6 where *n* represents animals). No significant differences were identified through a repeated measures one‐way ANOVA (all pairwise *P*‐values ≥0.093; exact values shown in Table 1). Abbreviations: endo, endocardium; epi, epicardium; LV, left ventricle; RV, right ventricle.

**FIGURE 2:**
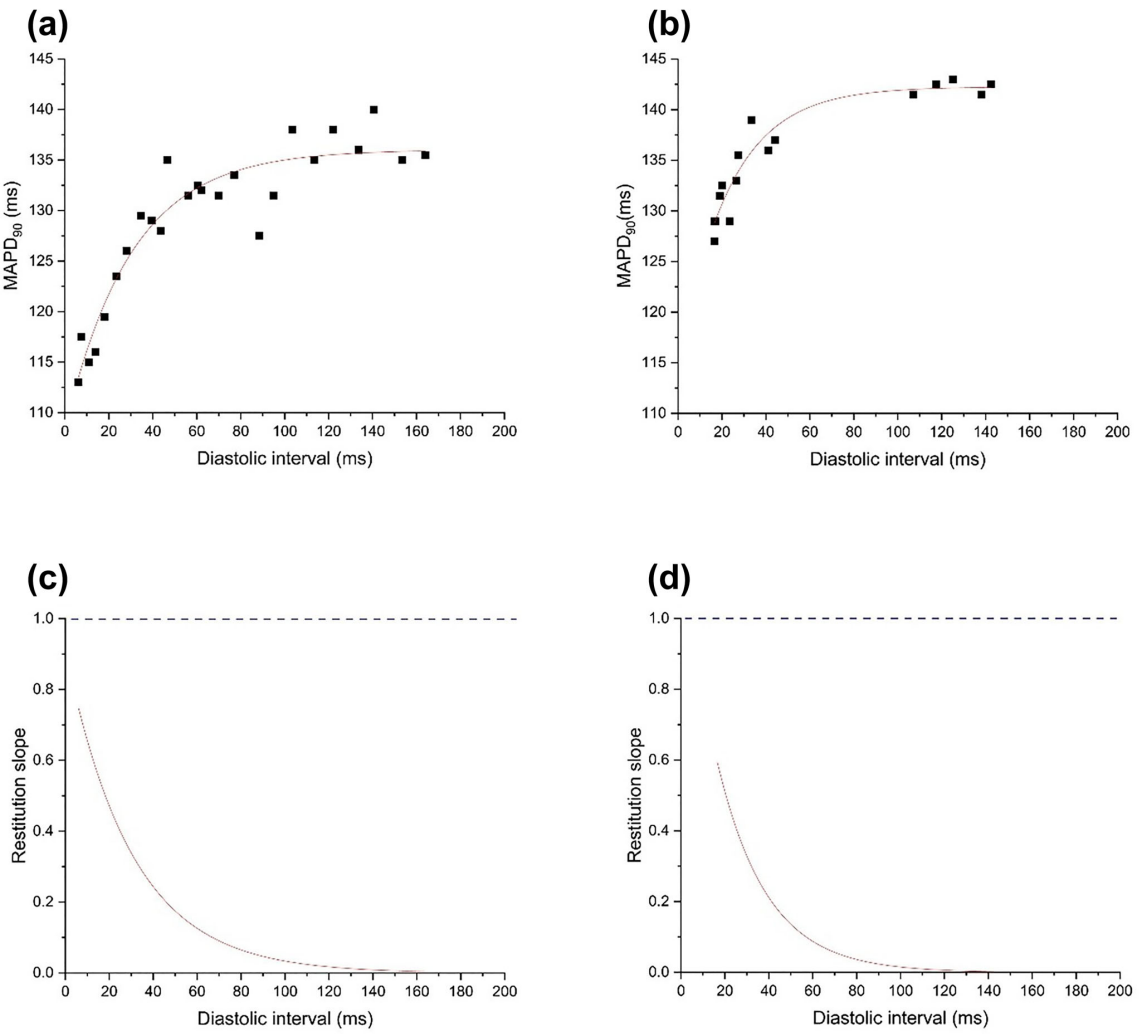
Plot of representative standard restitution curves for apex and base MAP contact electrodes. (a, b) Restitution curves (apex, a, and base, b) calculated from MAPD_S_2_ vs. preceding diastolic intervals (DI). The action potential duration restitution (APDR) curve (a, b) is fitted to the exponential curve [MAPD_90_ = MAPD_90max_(1 − e^−DI/τ)^]. (c, d) Derivative curve (apex, c, and base, d) produced from restitution data from to calculate APDR_max_ as shown by the red ×. APDR_max_ value of 0.75 (apex) and 0.59 (base). A horizontal dashed line is plotted at slope = 1.

**TABLE 1:** *P*‐values produced from pairwise comparisons of APD_90_ (MAPD_90_) and APDR_max_ between stimulation electrode sites.

Pacing sites being compared	*P*			
	Apical post‐pacing APD (Figure 1b)	Basal post‐pacing APD (Figure 1c)	Apical APDR (Figure 1d)	Basal APDR (Figure 1e)
RV apex endo vs. RV apex epi	0.856	0.998	0.945	0.816
RV apex endo vs. RV base epi	0.856	0.296	0.211	0.664
RV apex endo vs. LV base epi	0.991	0.957	0.998	0.464
RV apex endo vs. LV apex epi	0.999	0.525	0.553	0.995
RV apex epi vs. RV base epi	0.997	0.093	0.566	0.999
RV apex epi vs. LV base epi	0.977	0.924	0.901	0.670
RV apex epi vs. LV apex epi	0.812	0.345	>0.999	0.986
RV base epi vs. LV base epi	0.996	0.357	0.547	0.583
RV base epi vs. LV apex epi	0.204	>0.999	0.240	0.451
LV base epi vs. LV apex epi	0.982	0.440	0.945	0.303

*Note: P*‐values were derived from a repeated‐measures one‐way ANOVA followed by Tukey's *post hoc* multiple comparisons across all combinations of right and left ventricular (RV, LV) endocardial and epicardial stimulation sites. Comparisons are reported for post‐pacing APD_90_ measured at apical (Figure 1b) and basal (Figure 1c) recording sites, and APDR_max_ measured at apical (Figure 1d) and basal (Figure 1e) MAP contact electrodes. No statistically significant differences were observed between stimulation sites (all *P* ≥ 0.093; exact adjusted *P*‐values shown). Values represent Tukey‐adjusted *post hoc P*‐values following ANOVA. Abbreviations: APD_90_, action potential duration at 90% repolarization; APDR_max_, maximal action potential duration restitution slope; endo, endocardium; epi, epicardium; LV, left ventricle; MAPD_90_, monophasic action potential duration at 90% repolarization; RV, right ventricle.

### 3.2 The effect of pacing location on APDR_max_

The effect of altering pacing location on APD restitution from our experiments is illustrated in Figure 1d,e (exact *P*‐values shown in Table 1) with representative resitution curves found in Figure 2. APDR_max_ values when recording at the apex ranged from 0.49 ± 0.34 when pacing from the RV base epi to 0.93 ± 0.21 when pacing from the LV apex epi. Similar APDR_max_ values from the apex MAP recording site were produced from RV apex endo (0.70 ± 0.29), RV apex epi (0.91 ± 0.61) and LV base epi (0.76 ± 0.51). At the base recording site, APDR_max_ values were 1.44 ± 0.82 during LV base epi pacing, 0.62 ± 0.20 during LV apex epi pacing, 0.79 ± 0.22 during RV base epi pacing and 0.74 ± 0.54 during RV apex epi pacing. The maximal slope steepness when stimulating at the RV apex endo was similar from both contact electrode locations with a mean of 0.70 ± 0.29 from the apex and 0.57 ± 0.46 from the base recordings. There were no significant differences in APDR_max_ between pacing sites (all *P* ≥ 0.211).

### 3.3 The effects of different pacing locations on S_1_ and S_2_ delay

The results showed a significant increase in S_1_ delay at the apex MAP as illustrated in Figure 3, Appendix Figure A3 and Table 2. S_1_ delay was shorter when the pacing and recording sites were in closer proximity and longer when they were further apart. Specifically shorter S_1_ delays were observed when recording at the MAP apex between the pacing sites LV apex epi (20.7 ± 5.0 ms) and RV apex epi (36.5 ± 7.6 ms *P* = 0.045), LV base epi (40.6 ± 12.3 ms *P* = 0.010) and RV base epi (57.1 ± 16.1 ms *P* = 0.003). There is also a significant increase in S_1_ delay from the apical recordings between the basal stimulation locations (LV base and RV base *P* = 0.010). The same increase in S_1_ delay when moving further away from the recording site can be seen when comparing the base recordings and pacing site as illustrated in Figure 3 and Appendix Figure A3. Significant differences in S_1_ delay are seen between LV base (24.3 ± 8.7 ms) and LV apex epi (47.1 ± 6.8 ms *P* = 0.029), RV apex epi (53.5 ± 4.7 ms *P* = 0.002), and RV apex endo (51.0 ± 11.8 ms *P* = 0.003).

**FIGURE 3:**
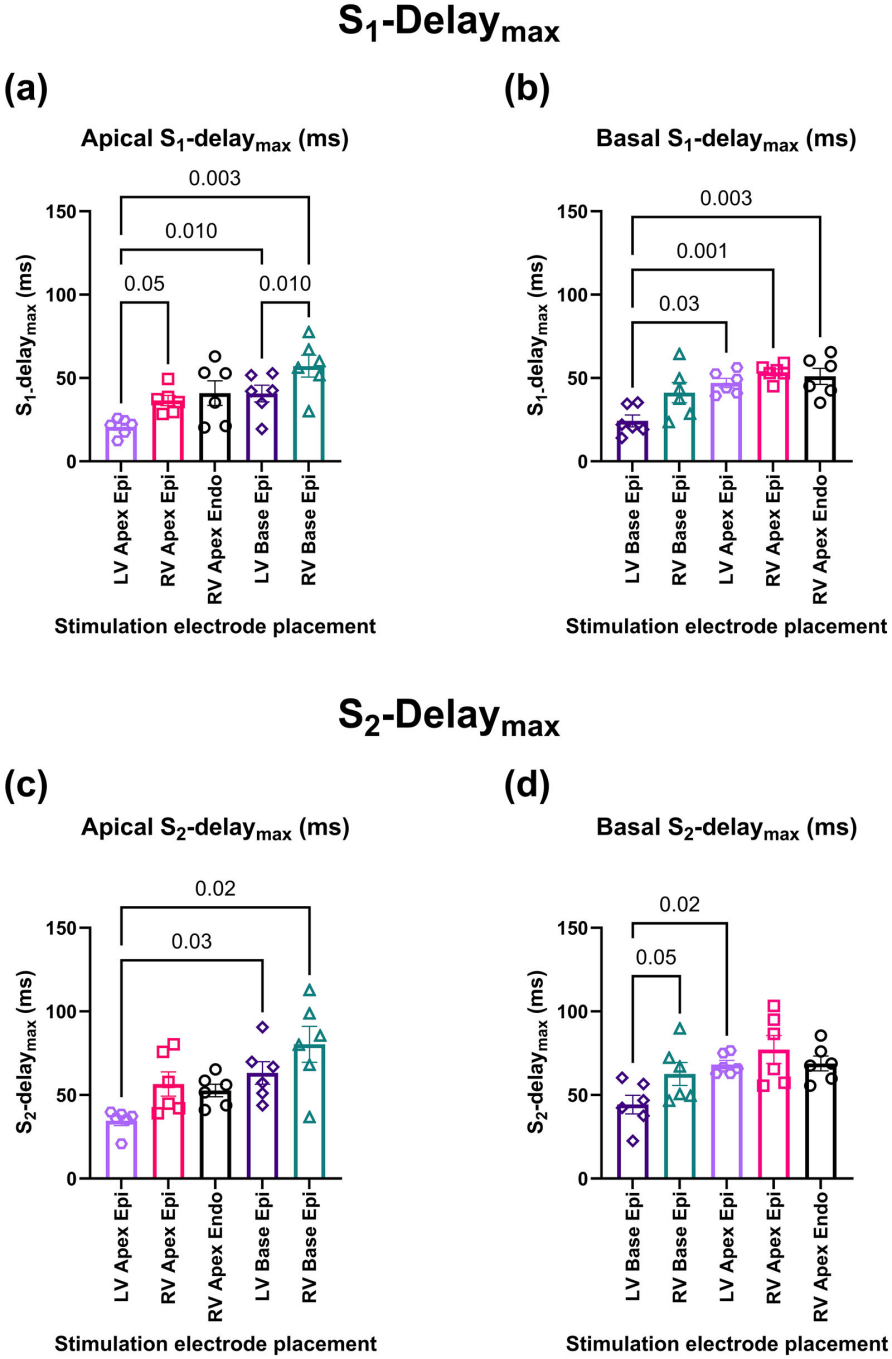
Effect of stimulation location on the delay between the stimuli and the start of the recorded action potential. (a, b) The S_1_ delay as calculated from the S_1_‐stimulus to the beginning of S_1_‐MAP signal from the apical (a) and basal (b) MAP contact electrode. (c, d) The S_2_ delay as calculated from the S_2_‐stimulus to the beginning of S_2_‐MAP signal from the apical (c) and basal (d) MAP contact electrode. Individual values are plotted with bars representing means ± SD (*n* = 6). Significant differences were identified through a one‐way repeated measure ANOVA with significant *P*‐values stated to three significant figures.

**TABLE 2:** *P*‐values for comparisons of stimulus‐to‐activation delay between stimulation locations (Figure 3).

Pacing sites being compared	*P*			
	S_1_‐delay_max_		S_2_‐delay_max_	
	Apical (Figure 3a)	Basal (Figure 3b)	Apical (Figure 3c)	Basal (Figure 3d)
RV apex endo vs. RV apex epi	0.956	0.980	0.982	0.912
RV apex endo vs. RV base epi	0.484	0.622	0.244	0.936
RV apex endo vs. LV base epi	>0.999	**0.003**	0.769	0.111
RV apex endo vs. LV apex epi	0.172	0.9237	0.082	>0.999
RV apex epi vs. RV base epi	0.176	0.324	0.184	0.567
RV apex epi vs. LV base epi	0.949	**0.002**	0.927	0.075
RV apex epi vs. LV apex epi	**0.045**	0.305	0.122	0.882
RV base epi vs. LV base epi	**0.010**	0.056	0.311	**0.05**
RV base epi vs. LV apex epi	**0.003**	0.933	**0.019**	0.914
LV base epi vs. LV apex epi	**0.010**	**0.029**	**0.032**	**0.018**

*Note: P*‐values correspond to comparisons of S_1_ and S_2_ stimulus‐to‐MAP onset delays between apical and basal MAP contact electrodes, as shown in Figure 3. Statistical analysis was performed using a one‐way repeated‐measures ANOVA. Individual comparisons are reported as adjusted *post hoc* values, with significance defined as *P* ≤ 0.05. Exact adjusted *P*‐values are provided with significant values in bold. Values represent Tukey‐adjusted *post hoc P*‐values following ANOVA. Abbreviations: endo, endocardium; epi, epicardium; LV, left ventricle; RV, right ventricle.

The study also analysed the relationship between S_2_ delay and pacing location. The results showed a significant increase in S_2_ delay when recording from the apex MAP between pacing sites LV apex epi (34.6 ± 7.0 ms) and LV base epi (63.2 ± 16.5 ms *P* = 0.032) and RV base epi (80.3 ± 26.3 ms *P* = 0.019). A similar increase in S_2_ delay can be seen when comparing the base recordings and pacing sites with significant differences in S_2_ delay being seen between LV base (44.3 ± 13.7 ms) and RV base epi (62.6 ± 16.9 ms *P* = 0.05), and LV apex epi (68.2 ± 10.9 ms *P* = 0.018). The largest difference when recording from the base was seen between LV base and RV apex epi (77.17 ± 20.4 ms *P* = 0.075).

### 3.4 The effects of different pacing locations on ERP

The mean ventricular ERP was similar for each pacing location (RV apex endo: 136.1 ± 16.1 ms, RV Apex epi: 120.8 ± 17.8 ms, RV Base epi: 130.4 ± 18.5 ms, LV Apex epi: 132.9 ± 9.8 ms and LV Base epi: 131.7 ± 8.6 ms) (Figure 4a and Table 3). The average ERP for all pacing locations was between 120–135 ms with no significant difference identified.

**FIGURE 4:**
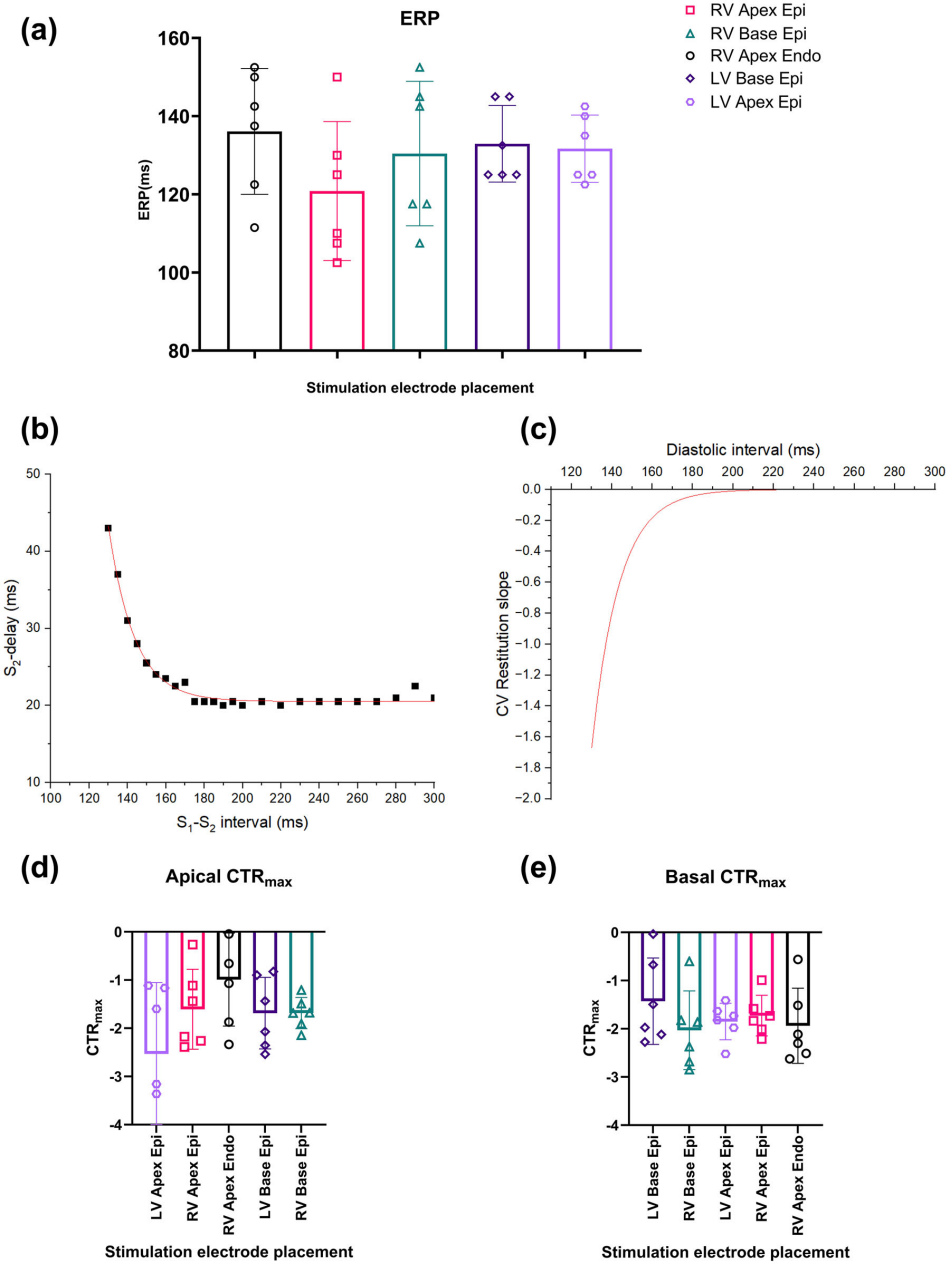
Effect of stimulation electrode placement on effective refractory period (ERP) and conduction time restitution max (CTR_max_). (a) The effective refractory period (ERP) for individual values plotted for each electrode placement. (b, c) The conduction time restitution (CVTR) curve (b) fitted to the exponential curve [S_2_‐delay = S_2_‐delaymax(1−e^−S1–S2interval/τ^)] and derivative curve (c) were produced from the apical contact electrode when pacing from the left ventricle apex epi. CTR_max_ is shown by the red × has a value of −1.67. (d, e) The CTR_max_ from the apical (d) and basal (e) MAP contact electrode are plotted for each electrode placement. Individual values are plotted with bars representing means ± SD (*n* = 6). No significant differences were identified through an ANOVA. Abbreviations: endo, endocardium; epi, epicardium; LV, left ventricle; RV, right ventricle.

**TABLE 3:** *P*‐values for comparisons of effective refractory period (ERP) and conduction time restitution (CTR_max_) between stimulation sites (Figure 4).

Pacing sites being compared	*P*‐values		
	ERP (Figure 4a)	Apical CTR (Figure 4d)	Basal CTR (Figure 4e)
RV apex endo vs. RV apex epi	0.400	0.796	0.974
RV apex endo vs. RV base epi	0.962	0.501	1.000
RV apex endo vs. LV base epi	0.996	0.791	0.616
RV apex endo vs. LV apex epi	0.985	0.531	0.999
RV apex epi vs. RV base epi	0.792	0.999	0.821
RV apex epi vs. LV base epi	0.621	1.000	0.902
RV apex epi vs. LV apex epi	0.710	0.630	0.985
RV base epi vs. LV base epi	0.998	>0.999	0.678
RV base epi vs. LV apex epi	1.000	0.727	0.976
LV base epi vs. LV apex epi	1.000	0.718	0.614

*Note: P*‐values correspond to comparisons of ERP, apical CTR_max_ and basal CTR_max_ across all stimulation electrode placements, as presented in Figure 4. Statistical analysis was performed using a one‐way repeated‐measures ANOVA followed by Tukey's *post hoc* multiple comparisons test. ERP values are shown in Figure 4a, while CTR_max_ values are shown for apical Figure 4d) and basal (Figure 4e) MAP contact electrodes. No statistically significant differences were observed between stimulation sites for ERP or CTR_max_ (all adjusted *P* ≥ 0.05) Exact adjusted *P*‐values are provided. Values represent Tukey‐adjusted *post hoc P*‐values following ANOVA. Abbreviations: CTR_max_, maximal conduction time restitution; epi, epicardium; endo, endocardium; ERP, effective refractory period; LV, left ventricle; RV, right ventricle.

### 3.5 The effects of different pacing locations on CTR_max_

The effect of altering pacing location on CTR_max_ restitution from our experiments is illustrated in Figure 4d,e and Table 3. The maximal slope is determined from a restitution curve as seen in Figure 4b,c and Table 3. The least steep maximal restitution slope (apex) was seen when pacing from the RV apex endo producing the lowest APDR_max_ value (−1.0 ± 1.0). The most steepened maximal slope mean (apex) was identified when pacing from the LV apex epi at −2.5 ± 1.5. A similar maximal restitution slope steepness was produced from the remaining pacing sites (MAP apex) with CTR_max_ mean values of RV apex epi (−1.6 ± 0.8), RV base epi (−1.7 ± 0.3) and LV base epi (−1.7 ± 0.7). The lowest CTR_max_ from the base MAP were seen from the LV base epi (−1.4 ± 0.9). The CTR_max_ values produced from the remaining pacing locations were similar: RV base epi (−2.0 ± 0.8), LV apex epi (−1.9 ± 0.4), RV apex epi (−1.7 ± 0.4) and RV apex endo (−1.9 ± 0.8). Overall, CTR_max_ values were comparable between pacing sites at both recording locations, and no significant differences were observed.

### 3.6 The relationship between APD and DI during VF

A negative linear relationship was identified between DI and APD_50_ during VF within the first 30 beats up to DI_(_ *_n_*_−5)_ (Figures 5, 6, Appendix Figures A4, A5, A6, A7, A8, A9 and Table 4). Outlier APD values >600 ms were excluded prior to statistical analysis to avoid distortion of correlation estimates. The negative relationship then deteriorates post‐30 beats with little to no relationship being seen at 90 beats post VF initiation (Figure 6). There is an increased negative relationship when analysing VF recordings from the apex contact MAP compared to the base recordings. The steepest negative relationship was identified from the apex recordings at 30 beats *R* = −0.5 where *R* represents the association between DI_(_ *_n_*_−1)_ and APD_50_. The negative relationship is maintained at similar *R*‐values (∼−0.2) for 30 beats when considering the basal recordings.

**FIGURE 5:**
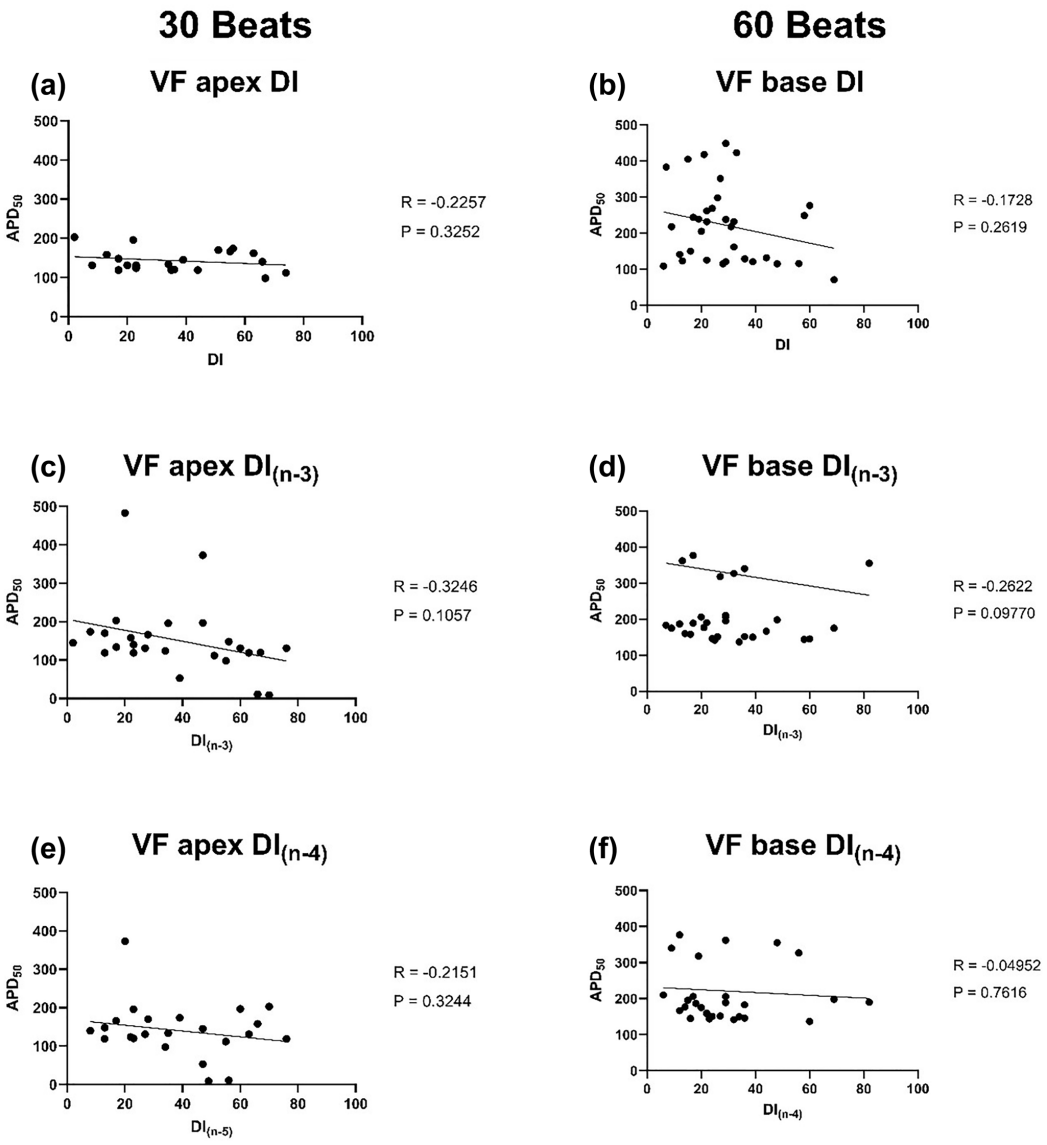
Representative plots showing the relationship between APD_50_ and previous (up to *n* − 5) diastolic intervals for either the first 30 (a, c, e) or the first 60 beats (b, d, f) following VF initiation. VF scatter plots from calculated APD_50_ (derived from the last normal action potential prior to VF initiation) and preceding diastolic intervals (DI) (a, b), DI_(_ *_n_*_−3)_ (c, d) and DI_(_ *_n_*_−5)_ (e, f) calculated from the *T*_act50_ and *T*_50_. The apical monophasic action potential (MAP) electrode data from an experiment conducted on the 27/2/22 for the 30 beats data. The basal MAP electrode data from an experiment conducted on the 8/3/22 for the 60 beats data. Both the *R*‐ and *P*‐value are shown as calculated by a simple linear regression using GraphPad Prism.

**FIGURE 6:**
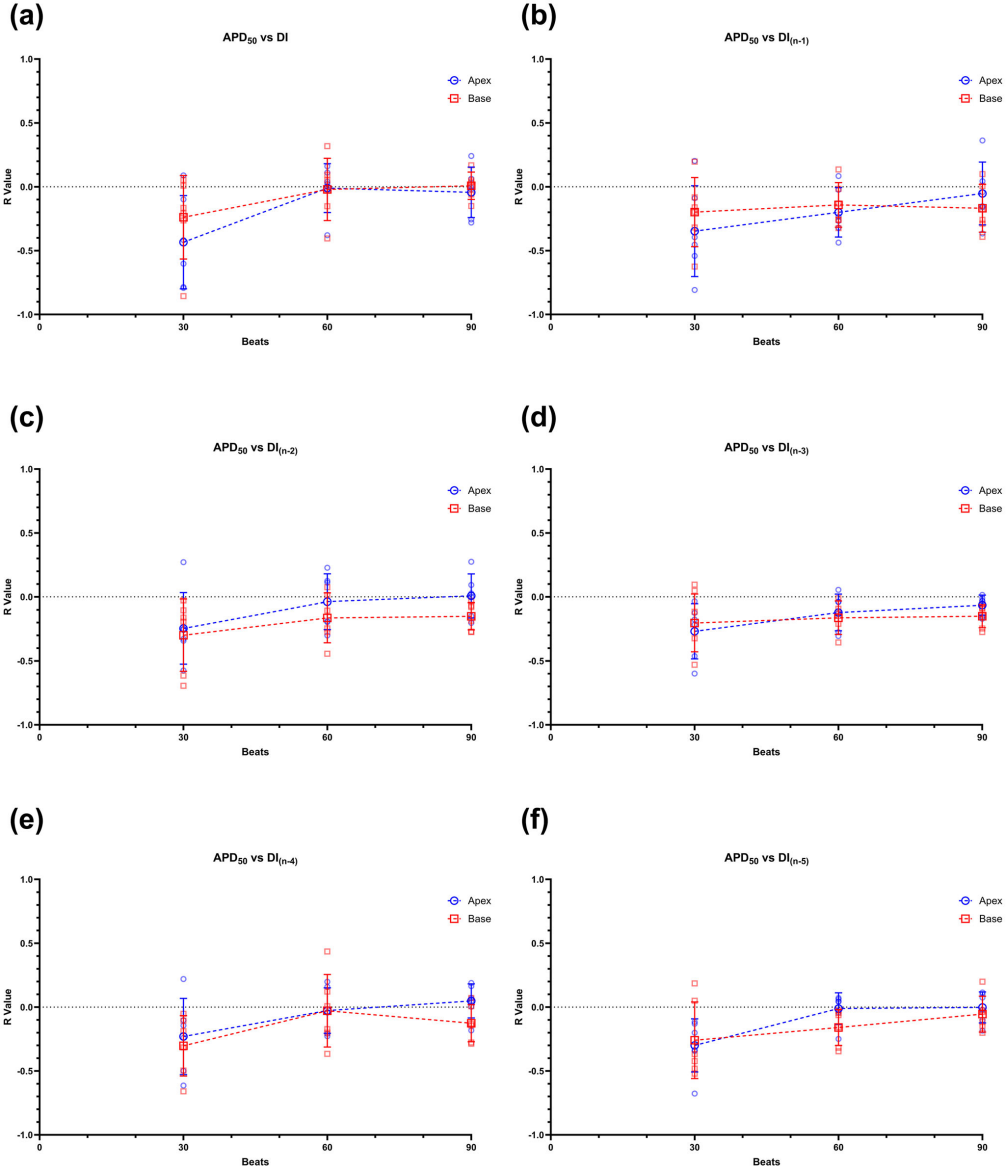
The relationship between APD_50_ and preceding DI (up to *n* − 5) from ventricular fibrillation (VF) initiation to 90 beats following. (a) The slope (*R*‐value) produced from a simple linear regression (Figure 7) between APD_50_ and preceding DI for the following 30, 60 and 90 beats from VF initiation. (b–e) The same information is plotted for APD_50_ vs. DI_(_ *_n_*_−2)_ (b), DI_(_ *_n_*_−3)_ (c), DI_(_ *_n_*_−4)_ (d) and DI_(_ *_n_*_−5)_ (e). Values plotted as means ± SD *n* = 6 with statistical analysis performed via two‐way ANOVA (Table 4) using GraphPad Prism.

**TABLE 4:** *P*‐values for comparisons of action potential duration 50 (APD_50_) and preceding APD_50_ up to *x* − 5 (Figure 6).

APD_50_ vs. DI							
		Apex			Base		
		30 beats	60 beats	90 beats	30 beats	60 beats	90 beats
Apex	30 beats		0.277	0.977	0.139		
	60 beats	0.277		0.198		0.358	
	90 beats	0.198	0.977				0.556
Base	30 beats	0.139				0.528	0.567
	60 beats		0.358		0.528		0.998
	90 beats			0.556	0.567	0.998	

APD_50_ vs. DI_(_ *_n_*_−1)_							
		Apex			Base		
		30 beats	60 beats	90 beats	30 beats	60 beats	90 beats
Apex	30 beats		0.502	0.790	0.560		
	60 beats	0.502		0.883		0.518	
	90 beats	0.790	0.883				0.935
Base	30 beats	0.560				0.977	0.998
	60 beats		0.518		0.977		0.989
	90 beats			0.935	0.998	0.989	

APD_50_ vs. DI_(_ *_n_*_−2)_							
		Apex			Base		
		30 beats	60 beats	90 beats	30 beats	60 beats	90 beats
Apex	30 beats		0.891	0.515	0.356		
	60 beats	0.891		0.792		0.880	
	90 beats	0.515	0.792				0.317
Base	30 beats	0.356				0.444	0.564
	60 beats		0.880		0.444		0.978
	90 beats			0.317	0.564	0.978	

APD_50_ vs. DI_(_ *_n_*_−3)_							
		Apex			Base		
		30 beats	60 beats	90 beats	30 beats	60 beats	90 beats
Apex	30 beats		**0.047**	**0.020**	0.525		
	60 beats	**0.047**		0.924		0.641	
	90 beats	**0.020**	0.924				0.628
Base	30 beats	0.525				0.113	0.053
	60 beats		0.641		0.113		0.927
	90 beats			0.628	0.053	0.927	

APD_50_ vs. DI_(_ *_n_*_−4)_							
		Apex			Base		
		30 beats	60 beats	90 beats	30 beats	60 beats	90 beats
Apex	30 beats		0.093	**0.034**	0.909		
	60 beats	0.093		0.887		0.076	
	90 beats	**0.034**	0.887				**0.018**
Base	30 beats	0.909				0.864	0.956
	60 beats		0.076		0.864		0.972
	90 beats			**0.018**	0.956	0.972	

APD_50_ vs. DI_(_ *_n_*_−5)_							
		Apex			Base		
		30 beats	60 beats	90 beats	30 beats	60 beats	90 beats
Apex	30 beats		0.051	0.110	0.428		
	60 beats	0.051		0.926		0.923	
	90 beats	0.110	0.926				0.490
Base	30 beats	0.428				0.226	0.137
	60 beats		0.923		0.226		0.957
	90 beats			0.490	0.137	0.957	

*Note: P*‐values derived from two‐way ANOVA performed on slope value (*R*) obtained from simple linear regression analyses of APD_50_ against DI up to *n* − 5. Comparisons include all combinations of recording location (apex, base) and VF duration from initiation (30, 60 and 90 beats). Each entry represents the *P*‐value for the corresponding pairwise comparison of *R*‐values between conditions. The table is arranged to distinguish within‐region (apex–apex, base–base) and between‐region (apex–base) comparisons. Results are shown for all datasets (*n* = 6). Significant *P*‐values are in bold.

Following the plotting of the APD_50_ and DI, further analysis was conducted to investigate the relationship between APD_50_ and preceding APD_50_ up to APD_50(_ *_x_*_−5)_ (Figures 7, 8, Appendix Figures A10, A11, A12, A13, A14, A15 and Table 5. A positive linear relationship is seen between APD_50_ and preceding APD_50_ up to (*x* − 5) within the first 30 beats (Figure 8). The highest *R*‐value was seen from the apex (0.4 ± 0.2) at 30 beats between APD_50_ versus APD_50(_ *_x_*_−1)_. The strength of the positive linear relationship decreases when looking at both the apex and base recordings post‐30 beats with little to no relationship being seen at 90 beats post‐VF initiation. The positive relationship seen at 30 beats is similar when comparing preceding APD_50_ from the basal MAP with *R*‐values ranging between 0.4 and 0.3. Following 60 beats post‐VF initiation, the *R*‐values for both apical and basal recordings decrease to ∼0.1 except for the base value produced when comparing APD_50_ versus APD_50(_ *_x_*_−1)_.

**FIGURE 7:**
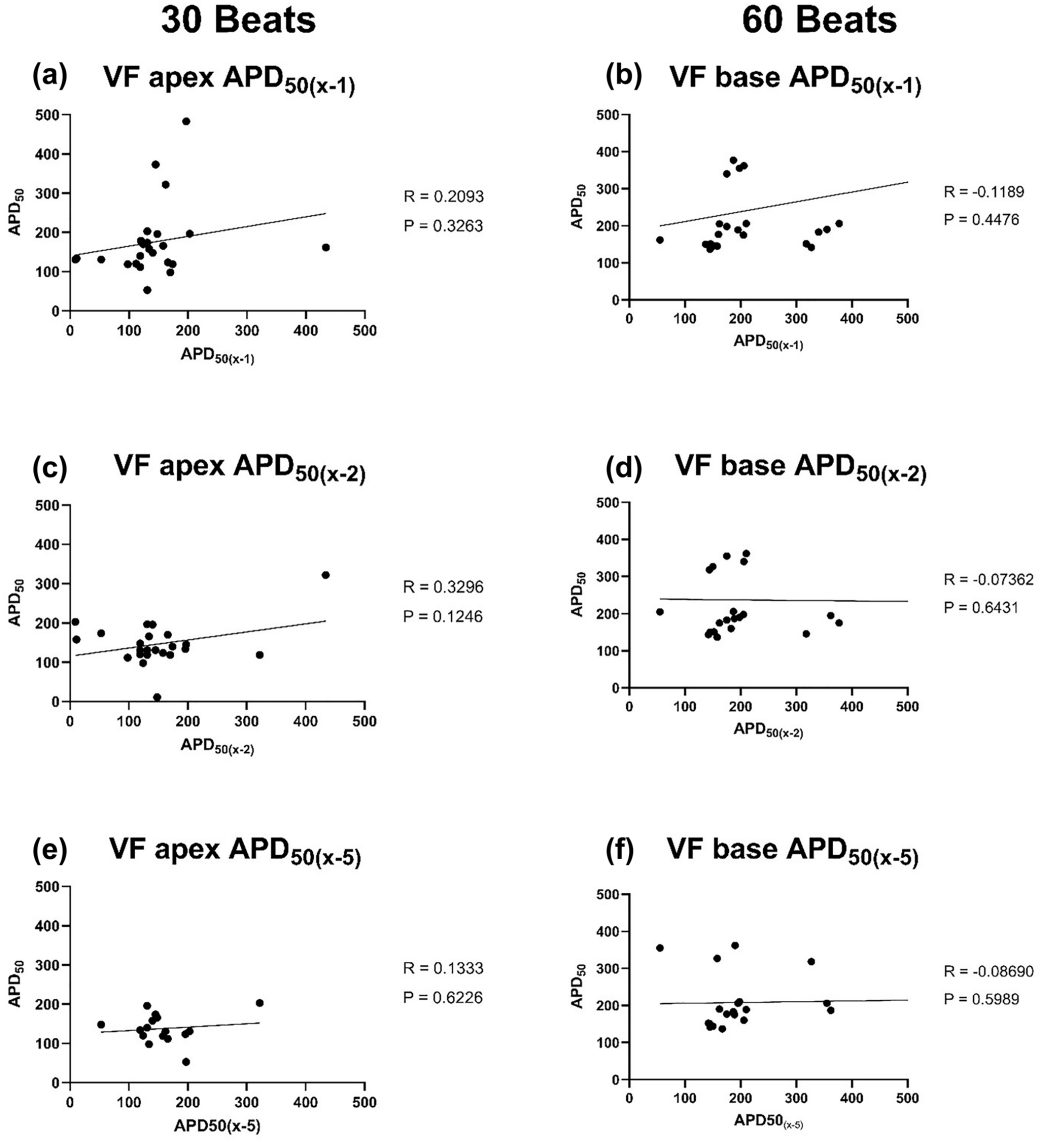
Representative plots showing the relationship between APD_50_ and previous APD_50_ (up to *x*−5) for either the first 30 (a, c, e) or 60 beats (b, d, f) following VF initiation. VF scatter plots from calculated APD_50_ (derived from the last normal action potential prior to VF initiation) and preceding APD_50(_ *_x_*_−1)_ (a, b), APD_50(_ *_x_*_−2)_ (c, d) and APD_50(_ *_x_*_−5)_ (e, f). The apical monophasic action potential (MAP) electrode data from an experiment conducted on the 27/2/22 for the 30 beats data. The basal MAP electrode data from an experiment conducted on the 8/3/22 for the 60 beats data. Both the *R*‐ and *P*‐value are shown as calculated by a simple linear regression using GraphPad Prism.

**FIGURE 8:**
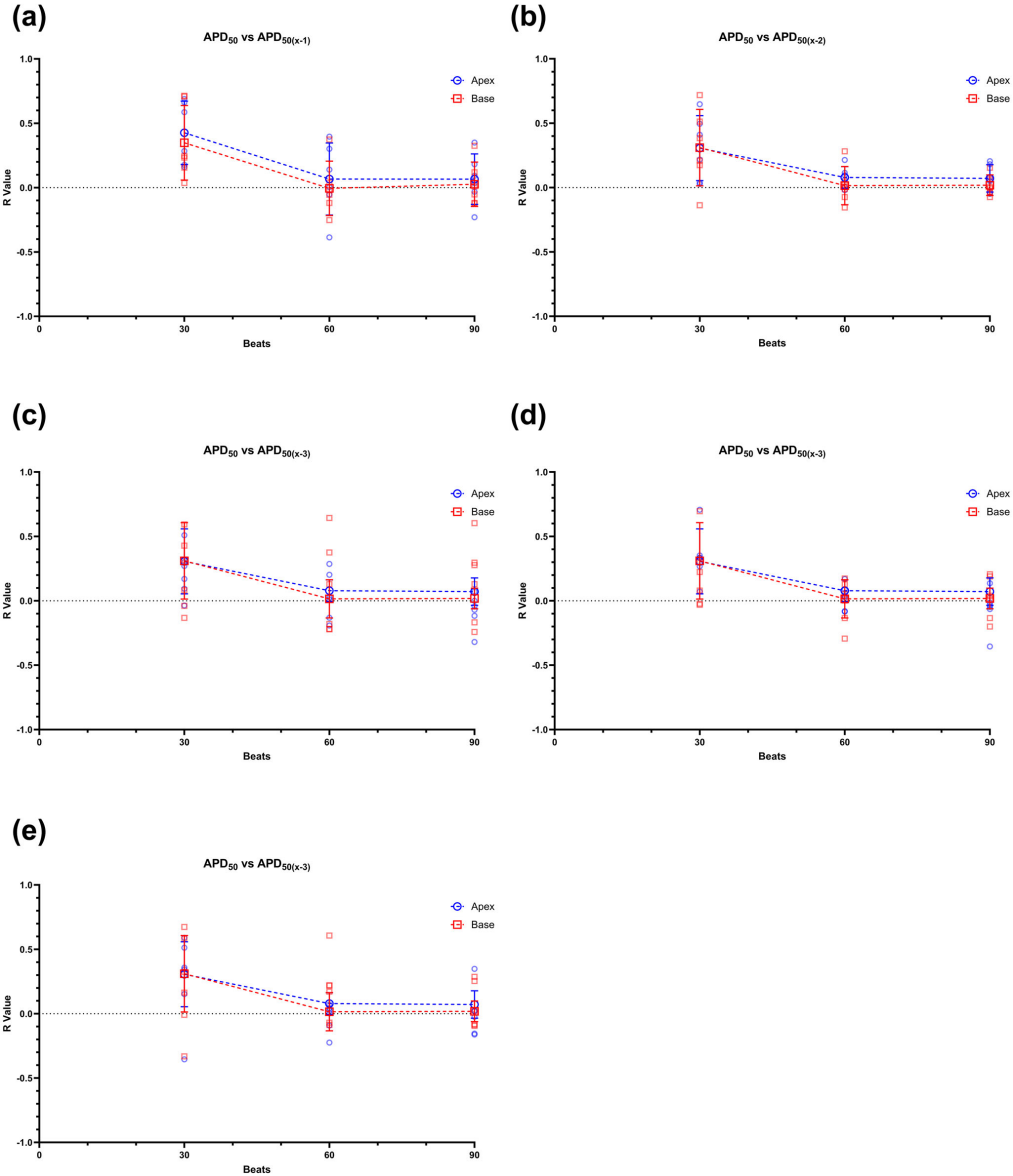
The relationship between APD_50_ and preceding APD_50_ (up to *x* − 5) from ventricular fibrillation (VF) initiation to 90 beats following. (a) The slope (*R*‐value) produced from a simple linear regression (Figure 7) between APD_50_ and preceding APD_50(_ *_x_*_−1)_ for the following 30,60 and 90 beats from VF initiation. (b–e) The same information is plotted for APD_50_ vs. APD_50(_ *_x_*_−2)_ (b), APD_50(_ *_x_*_−3)_ (c), APD_50(_ *_x_*_−4)_ (d) and APD_50(_ *_x_*_−5)_ (e). Values plotted as means ± SD, *n* = 6 with statistical analysis performed via two‐way ANOVA (Table 5) using GraphPad Prism.

**TABLE 5:** *P*‐values for comparisons of action potential duration 50 (APD_50_) and preceding APD_50_ up to *x* − 5 (Figure 8).

APD_50_ vs. APD_50(_ *_x_*_−1)_							
		Apex			Base		
		30 beats	60 beats	90 beats	30 beats	60 beats	90 beats
Apex	30 beats		0.127	0.280	0.070		
	60 beats	0.127		0.893		0.620	
	90 beats	0.280					0.984
Base	30 beats	0.070				0.870	0.927
	60 beats		0.620		0.870		0.990
	90 beats			0.984	0.927	0.990	

APD_50_ vs. APD_50(_ *_x_*_−2)_							
		Apex			Base		
		30 beats	60 beats	90 beats	30 beats	60 beats	90 beats
Apex	30 beats		0.457	0.526	0.896		
	60 beats	0.457		0.992		0.293	
	90 beats	0.526	0.992				0.710
Base	30 beats	0.896				0.080	0.526
	60 beats		0.293		0.080		0.655
	90 beats			0.710	0.526	0.655	

APD_50_ vs. APD_50(_ *_x_*_−3)_							
		Apex			Base		
		30 beats	60 beats	90 beats	30 beats	60 beats	90 beats
Apex	30 beats		0.284	0.094	0.761		
	60 beats	0.284		0.810		0.605	
	90 beats	0.094	0.810				**0.042**
Base	30 beats	0.761				0.093	0.735
	60 beats		0.605		0.093		0.346
	90 beats			0.042	0.735	0.346	

APD_50_ vs. APD_50(_ *_x_*_−4)_							
		Apex			Base		
		30 beats	60 beats	90 beats	30 beats	60 beats	90 beats
Apex	30 beats		**0.001**	**0.008**	0.293		
	60 beats	**0.001**		0.599		0.221	
	90 beats	**0.008**	0.599				0.976
Base	30 beats	0.293				0.220	0.115
	60 beats		0.221		0.220		0.932
	90 beats			0.976	0.115	0.932	

APD_50_ vs. APD_50(_ *_x_*_−5)_							
		Apex			Base		
		30 beats	60 beats	90 beats	30 beats	60 beats	90 beats
Apex	30 beats		0.189	0.125	0.772		
	60 beats	0.189		0.972		0.770	
	90 beats	0.125	0.972				0.718
Base	30 beats	0.772				0.190	0.141
	60 beats		0.770		0.190		0.985
	90 beats			0.718	0.141	0.985	

*Note*: P‐values derived from two‐way ANOVA performed on slope values (*R*) obtained from simple linear regression analyses of APD_50_ against APD_50_ up to *x* − 5. Comparisons include all combinations of recording location (apex, base) and VF duration from initiation (30, 60 and 90 beats). Each entry represents the *P*‐value for the corresponding pairwise comparison of *R*‐values between conditions. The table is arranged to distinguish within‐region (apex–apex, base–base) and between‐region (apex–base) comparisons. Results are shown for all datasets (*n* = 6) Significant *P*‐values are in bold.

## 4 DISCUSSION

This study investigated ventricular electrophysiology under two complementary conditions: controlled pacing at multiple ventricular sites, and early VF initiated from a single pacing location. Under controlled conditions, pacing location was used to assess its influence on steady‐state repolarisation, restitution properties, and conduction dynamics. In contrast, analysis of early VF was used to examine APD–DI relationships under dynamically varying activation, with a focus on short‐term, beat‐to‐beat cardiac memory.

Under stable pacing conditions we demonstrated that altering pacing location significantly affected S_1_ and S_2_ conduction delay, reflecting differences in activation sequence and propagation distance. However, steady‐state APD, APDR_max_, ERP and CTR_max_ were not significantly influenced by pacing location. In contrast, during ventricular fibrillation, APD exhibited a transient dependence on preceding DIs and preceding APDs, consistent with short‐term cardiac memory during the early phase of VF.

### 4.1 The influence of pacing location on steady‐state and dynamic repolarisation under stable activation conditions

Despite pacing from distinct ventricular locations, steady‐state APD did not differ significantly between pacing sites at either MAP location. Under conditions of constant pacing, the myocardium is exposed to a stable and repetitive activation history, allowing repolarisation to reach a steady electrophysiological state (Ng et al., 2001; Osadchii, 2012). In this state, APD appears to be determined primarily by intrinsic ion channel kinetics and electrotonic interactions, rather than by the spatial origin or direction of the activating wavefront (Eisner et al., 2009; Taggart & Lab, 2008). This absence of pacing‐site dependence is consistent with steady‐state conditions but does not preclude the possibility that longer‐term activation sequence‐dependent cardiac memory, which typically requires more prolonged pacing, was not engaged under the present experimental protocol. Long‐term activation sequence‐dependent cardiac memory was unlikely due to the short pacing duration. In the present study, pacing was performed only to establish steady‐state conditions prior to VF induction and was not maintained long enough to induce such longer‐term remodelling.

No significant differences in APD duration were observed when recording from or pacing at the apex, which may require further investigation as typically APDs are longer at the apex region then the base. This is due to a decrease in *I*_Ks_ and an increase in *I*_Kr_ within rabbit apical myocytes (Bartos et al., 2015). The direct mechanisms by which alternative pacing sites could influence APD, including pharmacological modulation or autonomic inputs, remain to be fully elucidated (Bartos et al., 2015; Chin et al., 2023; Ng, 2014; Ng et al., 2009).

Dynamic repolarisation properties were further examined using APDR. The APDR_max_ was also unaffected by pacing location. APDR_max_ values across all pacing sites were broadly consistent with trends reported in previous studies examining LV epicardial/endocardial and RV apex, septum and right atrial pacing (Ahlberg et al., 2007; Elliott et al., 2022; Osadchii, 2012). While these comparisons provide useful context, important species‐specific differences in cardiac electrophysiology should be considered. Ionic current expression varies substantially across species, particularly with respect to *I*_to_, which is minimal or absent in porcine and guinea pig myocardium, but contributes to early (phase 1) repolarisation in other species (Antzelevitch, 2000; Bartos et al., 2015). Rabbits also express ventricular *I*_to_; however, repolarisation is more strongly dependent on *I*_Kr_ and *I*_Ks_, with *I*_to_ contributing primarily to early repolarisation rather than the terminal phase of the ventricular action potential (Bartos et al., 2015; Ng et al., 2009). Additionally, species differences in intrinsic heart rate, action potential duration and restitution behaviour have been widely reported (Ahlberg et al., 2008; Osadchii, 2012). These factors influence both repolarisation dynamics and conduction behaviour and may affect the manifestation of restitution and cardiac memory. Therefore, direct quantitative comparisons between species should be interpreted with caution, and the present findings are best considered within the context of species‐specific electrophysiological properties. This is particularly relevant when considering extrapolation to human physiology, where repolarisation gradients and ion channel distributions differ further (Szentadrassy et al., 2005).

The rabbit ventricle shares several electrophysiological similarities with the human heart, including the presence of key repolarising currents such as *I*_Kr_ and *I*_Ks_; however, important differences exist that influence translational interpretation. In particular, rabbits exhibit a more prominent role of *I*_Kr_ in repolarisation and relatively reduced transmural heterogeneity compared with the human ventricle (Bartos et al., 2015). In humans, regional differences in APD and restitution are more strongly influenced by structural and fibre‐orientation‐dependent gradients (Antzelevitch, 2000; Szentadrassy et al., 2005), whereas in rabbits ionic current distribution is comparatively more uniform. These differences may affect both restitution slope behaviour and the spatial manifestation of cardiac memory, particularly under conditions of dynamic activation such as VF (Ahlberg et al., 2008; Osadchii, 2012; Pitruzzello et al., 2007). As a result, while rabbit models are highly useful for mechanistic electrophysiology, caution is required when extrapolating absolute restitution parameters or memory dynamics directly to human arrhythmogenesis.

Although previous studies have suggested that right‐sided pacing may increase VF susceptibility (Ahlberg et al., 2008; Wood et al., 1996), no such effect was observed in the present study, which may reflect differences in pacing protocol (extra stimulus protocol vs. dynamic pacing). Minor differences in APDR_max_ between pacing sites may reflect regional electrophysiological differences, although this cannot be directly confirmed in the present study. Further investigation into the differences between pacing site and arrhythmia susceptibility, namely epicardial versus RV apex endocardium, is important as RV apex endocardium is currently the standard pacing location clinically with ICDs also delivering electrical stimulation to the same location.

Although VF was not investigated directly, the data from this study suggest that the stability of local activation sequences, rather than the absolute pacing site, may play a greater role in influencing electrophysiological properties relevant to VF. Consistent with this, certain drugs that flatten the restitution curve have been shown to reduce VF susceptibility (Garfinkel et al., 2000), highlighting that the arrhythmogenic risk is shaped by the dynamic interplay of conduction properties rather than static location. Previous studies have reported an increase in VF susceptibility when comparing basal and apical stimulation sites (Choi & Salama, 2000; Pitruzzello et al., 2007), yet in our data, although some pacing locations produced slightly higher vulnerability, no significant differences were observed between apex and base. These findings suggest that differences previously attributed to pacing site may, at least in part, reflect variations in conduction distance and activation pathway length rather than intrinsic differences between apical and basal myocardium.

### 4.2 Influence of pacing location on conduction delays

While steady‐state repolarisation remained largely unaffected by pacing location, conduction delays following S_1_ and S_2_ stimulus demonstrated a clear dependence on spatial separation between pacing and recording site. Both S_1_ and S_2_ delays progressively increased as the distance between pacing site and recording location increased, highlighting the role of anatomical distance and local conduction properties in shaping electrophysiological responses.

These findings indicate that activation initiated near the recording location reaches the recording site more rapidly due to minimal conduction distance and preserved local properties, whereas remote pacing results in longer conduction times and accentuated delays. Importantly the S_2_ delay is particularly sensitive to local conduction slowing and gradients in refractoriness. Although APDR and S_2_ delay are derived from the same stimulus protocol, they interrogate distinct electrophysiological properties. APDR primarily reflects local repolarisation kinetics once activation has occurred (Osadchii, 2012; Pitruzzello et al., 2007; Qu et al., 1999; Wu & Patwardhan, 2004), whereas S_2_ delay reflects conduction of premature (S_2_) impulse through partially recovered myocardium, where reduced sodium channel availability and heterogeneities in refractoriness slow conduction and prolong activation time. As such, S_2_ delay captures conduction‐dependent effects that that are not reflected in restitution measurements, which primarily describes repolarisation behaviour following activation (Cao et al., 1999; Iravanian et al., 2023; Kléber, 2004; Lee et al., 2001; Prenner et al., 2016; Weiss et al., 1999).

The progressive increase in S_1_ and S_2_ delays with distance from pacing site reflects distance‐dependent conduction through ventricular tissue and is consistent with previously reported spatial variation in conduction properties (Bartos et al., 2015; Choi & Salama, 2000; Elliott et al., 2022; Osadchii, 2012; Pitruzzello et al., 2007; Wood et al., 1996). This functional divergence indicates that premature stimulation reveals pre‐existing spatial heterogeneities in conduction rather than altering intrinsic repolarisation kinetics. While these properties influence conduction during both S_1_ and S_2_ activation, their effects become more pronounced during premature stimulation, when incomplete recovery exaggerates regional differences in conduction. Therefore, the observed differences in S_2_ delay are consistent with spatial variation in conduction. However, because only single‐site MAP recordings were obtained, contributions from spatial heterogeneity in repolarisation or changes in wavefront propagation cannot be excluded. Because S_2_ stimuli are delivered during incomplete recovery, they are likely to accentuate pre‐existing regional differences in conduction and activation, thereby increasing sensitivity to spatial electrophysiological heterogeneity that is not apparent during steady‐state pacing. Instead, premature stimulation unmasks site‐dependent conduction heterogeneities because S_2_ stimuli are delivered during incomplete myocardial recovery, when regional differences in excitability and refractoriness have a greater influence on activation timing. Consequently, pre‐existing spatial differences in conduction between the pacing and recording sites become more apparent than during steady‐state pacing. This results in greater dispersion of activation timing under premature stimulation, which may contribute to arrhythmogenic mechanisms such as conduction block or re‐entry (Clayton & Bishop, 2014; Grune et al., 2021; Kléber, 2004; Lambiase et al., 2023). Importantly, although the dependence of activation delay on pacing‐recording distance is expected physiologically, the key observation is that this effect scaled consistently across multiple pacing sites without accompanying changes in APD, APDR_max_ or ERP. This dissociation suggests that pacing location primarily modulates conduction timing rather than intrinsic repolarisation properties under stable activation conditions.

Collectively, these findings demonstrate that ventricular activation is strongly governed by the spatial relationship between pacing and recording sites, with conduction delays scaling with distance while repolarisation properties remain stable. This dissociation indicates that pacing location predominantly influences conduction timing rather than intrinsic recovery of repolarisation. These results emphasise the importance of electroanatomic distance in shaping activation dynamics and suggest that observed differences in S_1_ and S_2_ delay primarily reflect conduction pathway length rather than intrinsic differences in arrhythmia susceptibility. Further studies should explore how and whether ischaemia, structural remodelling and fibrosis further alter these conduction dynamics (Ahlberg et al., 2008; Chattipakorn et al., 2005; Choi & Salama, 2000; Pitruzzello et al., 2007; Wood et al., 1996).

### 4.3 The impact of pacing location on CTR_max_

Following the analysis of S_1_ and S_2_ conduction delays, we assessed whether pacing location influenced CTR during the extra stimulus protocol. CT response was evaluated across S_2_ coupling intervals, allowing generation of CT restitution curves and calculation of the maximum conduction time restitution (CTR_max_). The results indicate that pacing location did not significantly alter conduction time response to premature stimulation (CTR_max_), suggesting that the dynamic response of conduction time to premature stimulation was preserved across pacing sites. While computational studies have suggested that reduced dependence of conduction time on diastolic interval (i.e. CT restitution flattening) may promote wavebreak and discordant CT alternans independent of APDR (Qu et al., 1999), no evidence of altered CT response to premature stimulation was observed across pacing sites. No specific ionic current measurements were performed; therefore, these mechanisms are inferred from established electrophysiological principles rather than directly measured. A modest increase in CTR_max_ was observed when recording from the apex MAP and pacing from the LV apex epi at −2.5 ± 0.6 ms; however, this did not reach statistical significance. CTR_max_ values were broadly similar amongst both apical and basal recordings. These findings indicate that although increasing the distance between pacing and recording locations significantly affected S_1_ and S_2_ activation delays, it does not substantially alter CTR_max_. The absence of a pronounced effect of pacing location on CTR_max_ may reflect preserved fast sodium current (*I*_Na_) availability, which governs conduction time, together with the contribution of the inward rectifier potassium current (*I*_K1_), which maintains resting membrane potential and excitability under premature stimulation (Qu et al., 1999).

### 4.4 The role of cardiac memory and restitution in ventricular fibrillation

Building on the impact of pacing location on conduction and repolarisation under premature stimulation, we next examined how cardiac memory and restitution dynamics influence early VF. It is important to distinguish between short‐term cardiac memory, as examined in this study, and classical activation sequence‐dependent memory. The latter develops over prolonged pacing and involves ion channel remodelling, whereas the former reflects beat‐to‐beat dependence of APD on recent activation history. Given the relatively short pacing durations used in this study, it is unlikely that activation sequence‐dependent memory mechanisms were significantly engaged. The activation sequence‐dependent form of cardiac memory, involving remodelling of ionic currents such as *I*_to_ and *I*_Ks_, requires sustained alterations in activation sequence over extended pacing periods. In this study, VF was induced following a short burst pacing protocol, after which pacing was terminated. As such, the experimental design did not include prolonged pacing or maintained altered activation sequences and therefore was not intended to induce or assess this form of cardiac memory.

Although both the S_1_–S_2_ extra‐stimulus protocol and the VF analysis examine APD–DI relationships, they represent fundamentally different electrophysiological conditions. The S_1_–S_2_ protocol characterises intrinsic restitution properties under controlled pacing, where activation sequence and cycle length are fixed. In contrast, during VF activation sequence, cycle length, wavefront direction and electrotonic interactions vary continuously. Consequently, the APD–DI relationships observed during VF should not be interpreted as classical restitution curves but rather as dynamic relationships that reflect both intrinsic restitution properties and short‐term activation history.

Previous studies in swine models have explored the relationship between APD and DI during VF (Huang et al., 2004) finding that a simple linear relationship did not adequately describe the data over prolonged VF. In contrast a modest linear relationship was evident primarily during the earliest phase of VF (approximately the first 30 beats) after which the association progressively weakened. This suggests that early VF exhibits somewhat structured, non‐random behaviour consistent with short‐term cardiac memory. These findings are also consistent with Toal et al. (2009), who reported negative APD–DI slopes during human VF, indicating that APD–DI dynamics during fibrillation differ from those predicted by classical restitution under controlled pacing conditions (Toal *et al.*, 2009). However, species‐specific differences in ionic current expression and repolarisation mechanism should be considered when interpreting these comparisons.

Our data indicates that DI is associated with APD during the initial VF beats, and that preceding APD is positively correlated with subsequent APD, supporting the presence of short‐term cardiac memory, similar to observations by Huang et al. (2004). Although a linear relationship is identifiable, the exact nature of these interactions remains unclear and warrants further investigation.

Although S_1_–S_2_ restitution properties were unaffected by pacing location, APD during early VF remained associated with both preceding DI and preceding APD. This suggests that intrinsic restitution alone does not fully explain repolarisation dynamics during fibrillation. Instead, activation history appears to contribute to APD behaviour during the early stages of VF.

In classical restitution theory, the maximal slope of the APD restitution curve is thought to influence VF maintenance, with slopes greater than 1 increasing the likelihood of wavebreak and sustained VF (Weiss et al., 1999). However, the APD–DI relationships examined during VF in the present study should not be interpreted as classical restitution slopes. During VF, activation sequence, cycle length and electrotonic interactions vary continuously, and the observed negative APD–DI relationships therefore reflect dynamic, history‐dependent repolarisation rather than intrinsic restitution alone. Within the first 30 beats, the negative linear relationship between APD and DI suggests that early VF dynamics are sensitive to short‐term changes in DI. Notably a negative linear relationship was observed when plotting APD against preceding DI up to *n* − 5, although its significance decreased for more distant preceding beats. This temporal evolution is consistent with the observations of Toal et al. (2009), who also reported negative APD–DI slopes during human VF, supporting the concept that APD–DI behaviour during fibrillation cannot be fully explained by classical restitution measured under controlled pacing conditions.

Understanding these early VF relationships may have clinical implications. If these early APD–DI relationships during VF can be better characterised, they may ultimately inform optimal shock delivery timing, potentially improving defibrillation efficacy. Further investigation is needed to clarify the exact interactions between APD and DI and to determine how early VF dynamics vary with activation history and recording location.

These species‐specific differences are also relevant to interpretation of cardiac memory mechanisms as well as also influencing VF‐related restitution and cardiac memory behaviour. In contrast, the human ventricle exhibits greater structural and electrophysiological heterogeneity, which may introduce additional spatial components to cardiac memory and modify restitution behaviour during rapid activation such as VF.

### 4.5 Limitations

This study has several limitations. Recordings were obtained using single‐site MAP contact electrodes, which provide high‐fidelity local signals but do not capture global activation patterns or spatial dispersion across the heart. In addition, APD measurement during VF is technically challenging due to rapid cycle length variation, signal noise and changes in repolarisation morphology. Although a consistent APD_50_ detection criterion was applied across all analysed beats, variability in repolarisation waveforms may introduce uncertainty in the precise determination of APD and DI values. However, the analysis focused on within‐preparation relationships between preceding DI/APD and subsequent APD rather than absolute APD values, reducing the impact of potential measurement variability on interpretation of temporal associations.

All measurements were confined to the left ventricle, limiting assessment of regional differences between ventricles and other cardiac regions. VF was induced from a single pacing site following a short burst protocol, so the influence of different pre‐VF activation sites on fibrillation dynamics could not be evaluated. In addition, the *ex vivo* Langendorff‐perfused rabbit heart model lacks neurohumoral and mechanical influences present in vivo, and species‐specific differences in ionic current expression limit direct translation to human physiology. Finally, analysis was restricted to early VF, and longer‐duration fibrillation dynamics were not assessed.

### 4.6 Conclusion

This study demonstrates that ventricular electrophysiology is largely robust to pacing location under steady‐state conditions, with minimal differences in APD, APDR_max_ and ERP across sites, suggesting the use of RV apex pacing as a representative measure of ventricular repolarisation. In contrast, local conduction delays are highly sensitive to the spatial relationship between pacing and recording sites, with both S_1_ and S_2_ delays increasing progressively with distance. These findings highlight the importance of activation sequence and conduction dynamics in shaping ventricular electrophysiological behaviour, beyond static repolarisation properties alone. During early VF, APD exhibited modest association with preceding DI and preceding APD, consistent with short‐term cardiac memory. Although S_1_–S_2_ extra‐stimulus protocol characterises the intrinsic restitution properties of the myocardium under controlled pacing conditions, these measurements did not fully explain the dynamic APD–DI relationships observed during VF, where activation history, continuously changing activation sequence and electrotonic interactions contribute to repolarisation behaviour. These findings should be interpreted in the context of species‐specific electrophysiological differences, which may influence their direct translation to human cardiac physiology. The structured APD–DI relationships observed during early VF suggest that a better understanding of activation history and dynamic repolarisation may ultimately inform intervention strategies such as pharmacological modulation of restitution, targeted pacing approaches or optimisation of ICD shock timing.

Overall, the results emphasise that while standard pacing locations capture baseline repolarisation, local conduction properties and activation history are critical determinants of arrhythmogenic risk, with important implications for both experimental design and clinical therapy. Further investigation into the interplay between APD, DI and conduction heterogeneity during VF may provide mechanistic insights to improve sudden cardiac death prevention.

## AUTHOR CONTRIBUTIONS

Bethan Roper‐Jones: Investigation, formal analysis, data curation, visualization, writing – original draft. Emily Allen: Supervision, validation, writing – review & editing. Rachel L. Sutcliffe: Formal analysis, writing – review & editing. G. André Ng: Funding acquisition, methodology, supervision. Reshma A. Chauhan: Project administration, supervision, writing – review & editing. All authors have read and approved the final version of this manuscript and agree to be accountable for all aspects of the work in ensuring that questions related to the accuracy or integrity of any part of the work are appropriately investigated and resolved. All persons designated as authors qualify for authorship, and all those who qualify for authorship are listed.

## CONFLICT OF INTEREST

The authors declare they have no conflicts of interest.

## Figures and Tables

**FIGURE A1:**
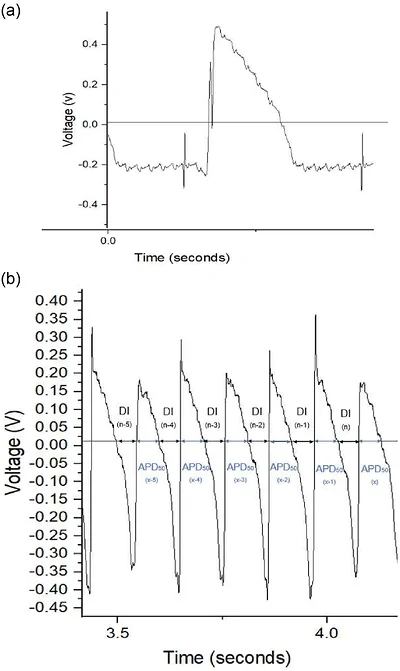
(a) The last action potential prior to ventricular fibrillation (VF) with a line through the action potential duration 50 (APD_50_) as calculated. (b) The action potentials during ventricular fibrillation with the APD_50_ line. *T*_act50_ and *T*_50_ were calculated from the time each action potential crosses the APD_50_ line. The blue arrows indicate APD_50_ up to *x* − 5 and the black arrows indicate the DI up to *n* − 5.

**FIGURE A2:**
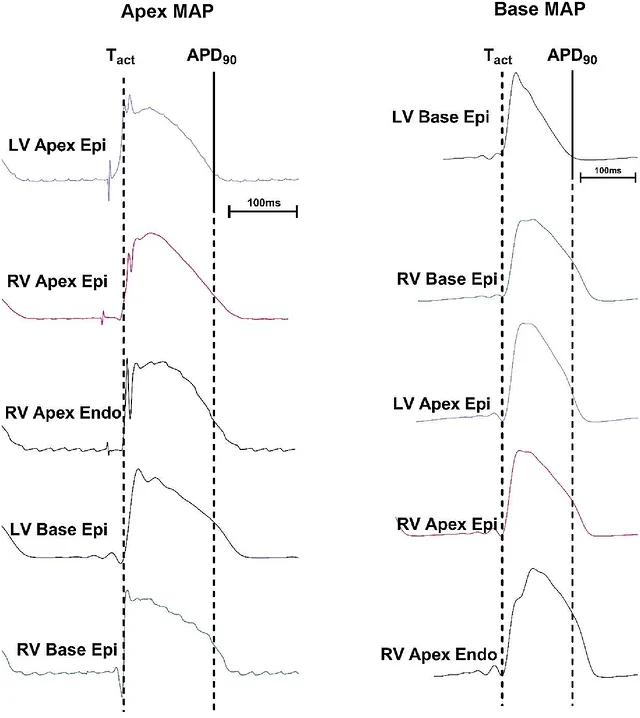
Monophasic action potentials (MAPs) during constant placing for each stimulation electrode placement for apical (left) and basal (right) contact electrodes. The MAPs are presented by distance from recording location in order from closest (top) to furthest away (bottom). The APD_90_ was calculated from the stimulation closest to the recording location – left ventricle (LV) apex for apex MAP and LV base for base MAP. The *T*_act_ has been lined up for each stimulation placement to allow for APD_90_ comparison. Abbreviations: endo, endocardium; epi, epicardium; LV, left ventricle; RV, right ventricle.

**FIGURE A3:**
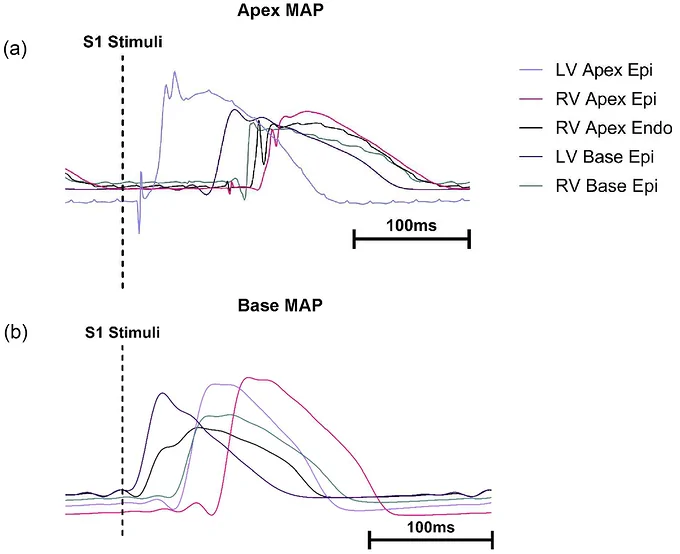
Monophasic action potentials (MAPs) following S_1_ stimuli for each stimulation electrode placement for apical (a) and basal (b) contact electrodes. Abbreviations: endo, endocardium; epi, epicardium; LV, left ventricle; RV, right ventricle.

**FIGURE A4:**
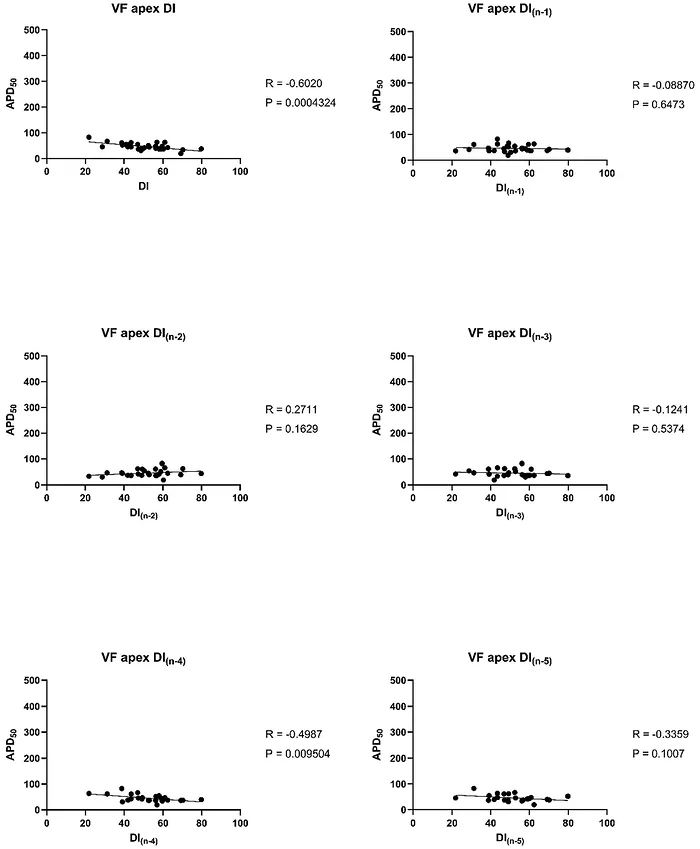
Representative plots showing the relationship between APD_50_ and previous (up to *n* − 5) diastolic intervals for the first 30 beats following VF initiation as recorded from the apical monophasic action potential (MAP) electrode from an experiment conducted on the 27/2/22. VF scatter plots from calculated APD_50_ (derived from the last normal action potential prior to VF initiation) and preceding diastolic intervals (DI up to *n* − 5) calculated from the *T*_act50_ and *T*_50_. Both the *R*‐ and *P*‐value are shown as calculated by a simple linear regression using GraphPad Prism.

**FIGURE A5:**
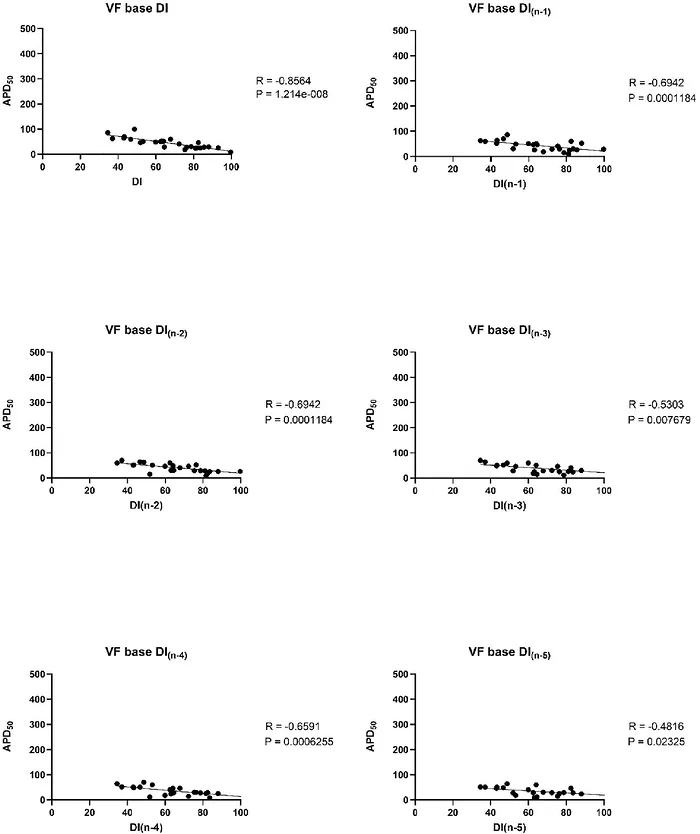
Representative plots showing the relationship between APD_50_ and previous (up to *n* − 5) diastolic intervals for the first 30 beats following VF initiation as recorded from the basal monophasic action potential (MAP) electrode from an experiment conducted on the 8/3/22. VF scatter plots from calculated APD_50_ (derived from the last normal action potential prior to VF initiation) and preceding diastolic intervals (DI up to *n* − 5) calculated from the *T*_act50_ and *T*_50_. Both the *R*‐ and *P*‐value are shown as calculated by a simple linear regression using GraphPad Prism.

**FIGURE A6:**
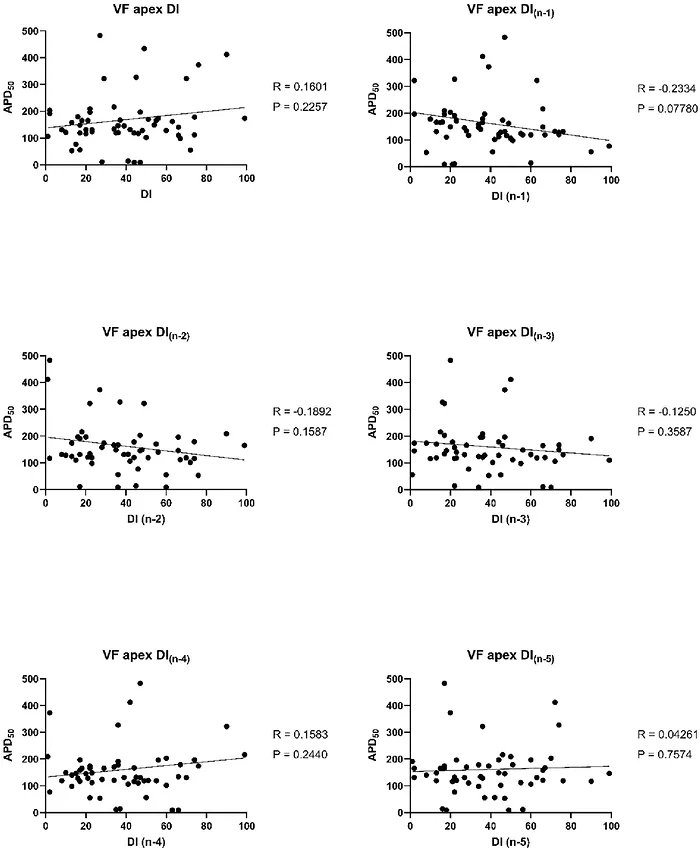
Representative plots showing the relationship between APD_50_ and previous (up to *n* − 5) diastolic intervals for the first 60 beats following VF initiation as recorded from the apical monophasic action potential (MAP) electrode from an experiment conducted on the 27/2/22. VF scatter plots from calculated APD_50_ (derived from the last normal action potential prior to VF initiation) and preceding diastolic intervals (DI up to *n* − 5) calculated from the *T*_act50_ and *T*_50_. Both the *R*‐ and *P*‐value are shown as calculated by a simple linear regression using GraphPad Prism.

**FIGURE A7:**
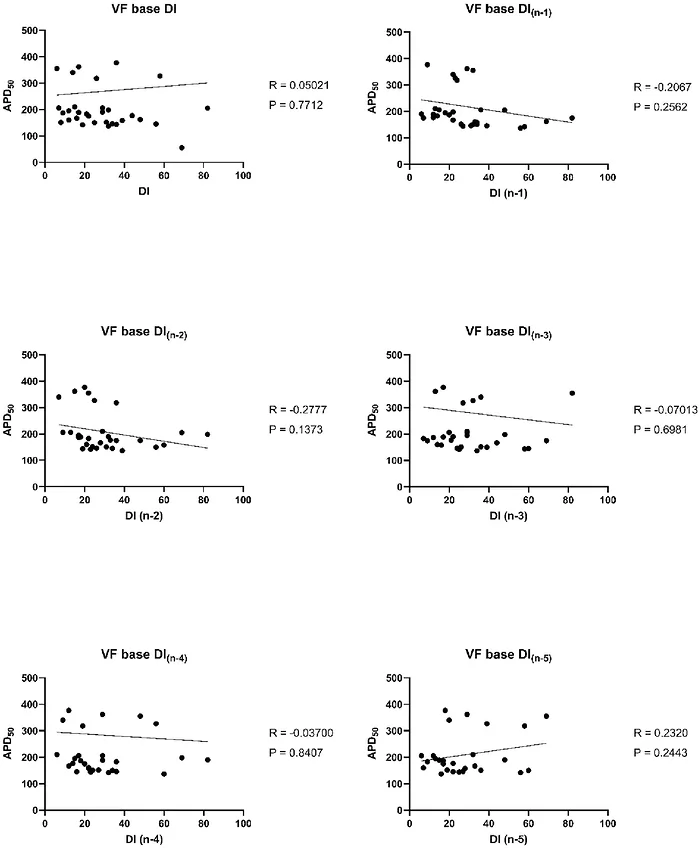
Representative plots showing the relationship between APD_50_ and previous (up to *n* − 5) diastolic intervals for the first 60 beats following VF initiation as recorded from the basal monophasic action potential (MAP) electrode from an experiment conducted on the 8/3/22. VF scatter plots from calculated APD_50_ (derived from the last normal action potential prior to VF initiation) and preceding diastolic intervals (DI up to *n* − 5) calculated from the *T*_act50_ and *T*_50_. Both the *R*‐ and *P*‐value are shown as calculated by a simple linear regression using GraphPad Prism.

**FIGURE A8:**
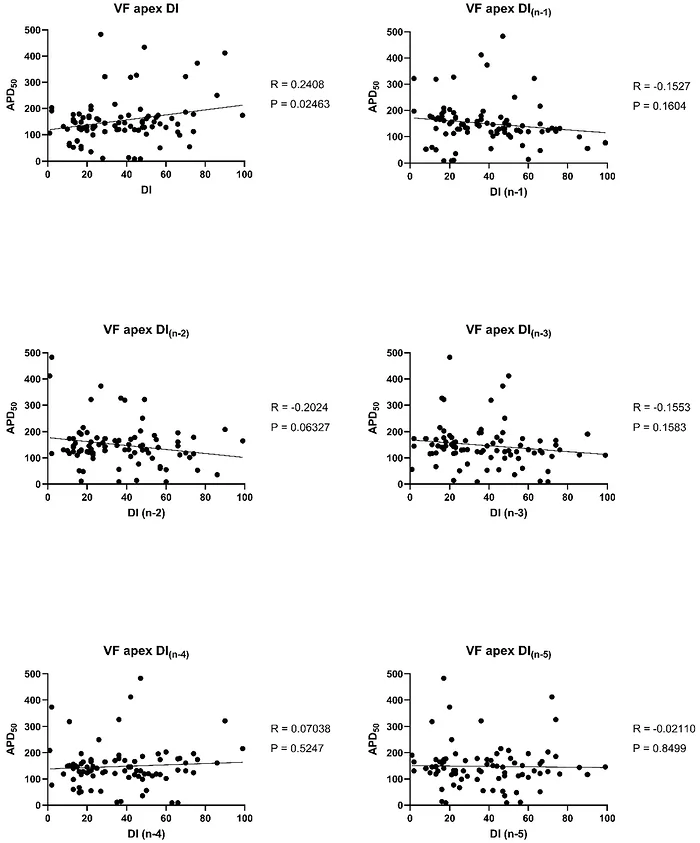
Representative plots showing the relationship between APD_50_ and previous (up to *n* − 5) diastolic intervals for the first 90 beats following VF initiation as recorded from the apical monophasic action potential (MAP) electrode from an experiment conducted on the 27/2/22. VF scatter plots from calculated APD_50_ (derived from the last normal action potential prior to VF initiation) and preceding diastolic intervals (DI up to *n* − 5) calculated from the *T*_act50_ and *T*_50_. Both the *R*‐ and *P*‐value are shown as calculated by a simple linear regression using GraphPad Prism.

**FIGURE A9:**
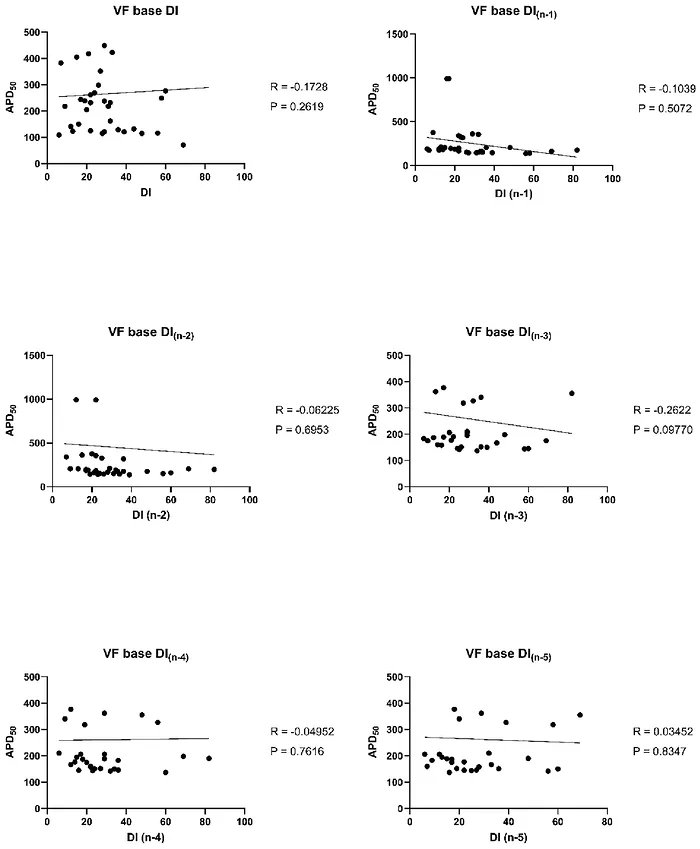
Representative plots showing the relationship between APD_50_ and previous (up to *n* − 5) diastolic intervals for the first 90 beats following VF initiation as recorded from the basal monophasic action potential (MAP) electrode from an experiment conducted on the 8/3/22. VF scatter plots from calculated APD_50_ (derived from the last normal action potential prior to VF initiation) and preceding diastolic intervals (DI up to *n* − 5) calculated from the *T*_act50_ and *T*_50_. Both the *R*‐ and *P*‐value are shown as calculated by a simple linear regression using GraphPad Prism.

**FIGURE A10:**
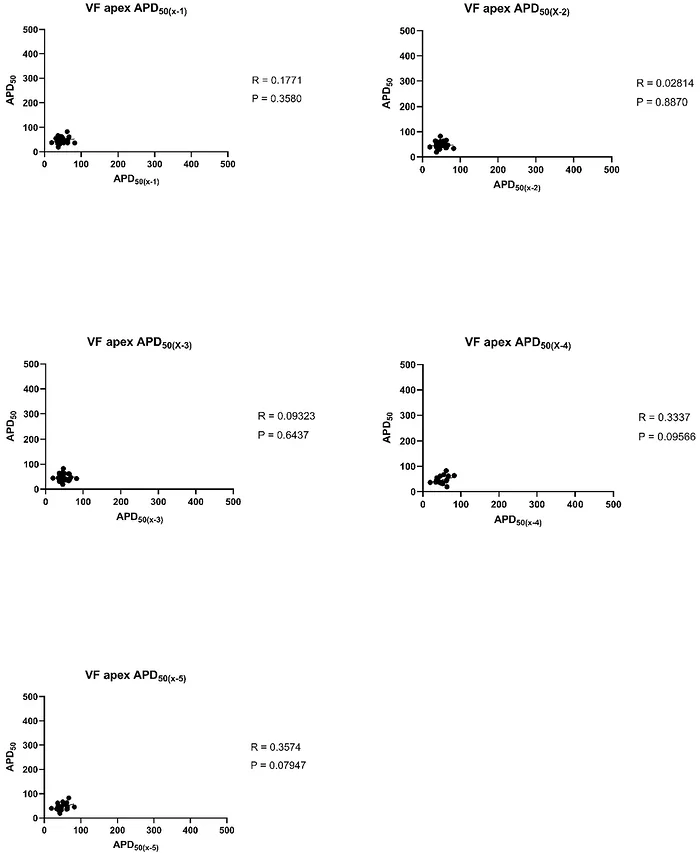
Representative plots showing the relationship between action potential duration (APD) 50 and previous (up to *x* − 5) APD for the first 30 beats following VF initiation as recorded from the apical monophasic action potential (MAP) electrode from an experiment conducted on the 27/2/22. VF scatter plots from calculated APD_50_ (derived from the last normal action potential prior to VF initiation) and preceding APD_50_ (up to *x* − 5) calculated from the *T*_act50_ and *T*_50_. Both the *R*‐ and *P*‐value are shown as calculated by a simple linear regression using GraphPad Prism.

**FIGURE A11:**
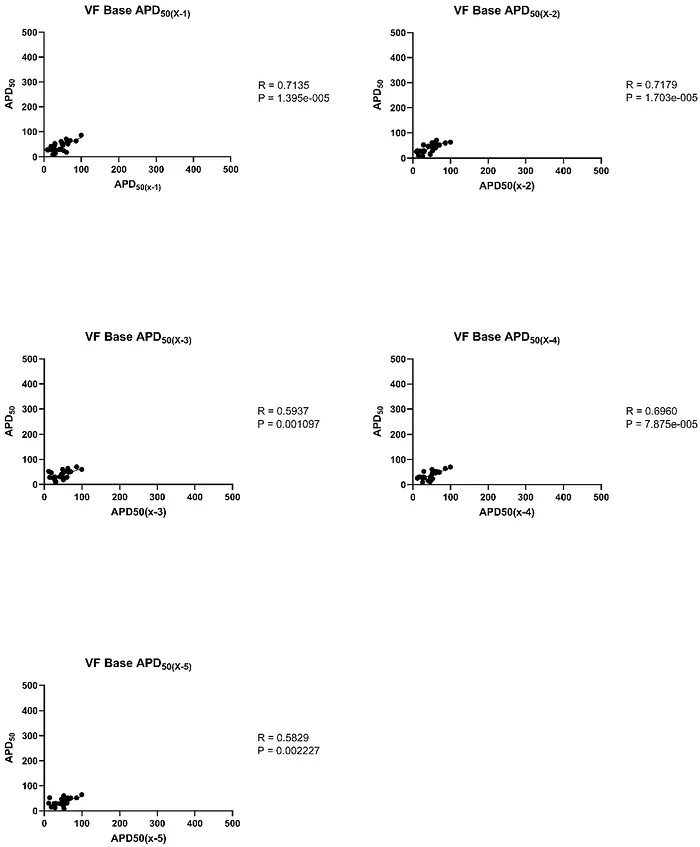
Representative plots showing the relationship between action potential duration (APD) 50 and previous (up to *x* − 5) APD for the first 30 beats following VF initiation as recorded from the basal monophasic action potential (MAP) electrode from an experiment conducted on the 8/3/22. VF scatter plots from calculated APD_50_ (derived from the last normal action potential prior to VF initiation) and preceding APD_50_ (up to *x* − 5) calculated from the *T*_act50_ and *T*_50_. Both the *R*‐ and *P*‐value are shown as calculated by a simple linear regression using GraphPad Prism.

**FIGURE A12:**
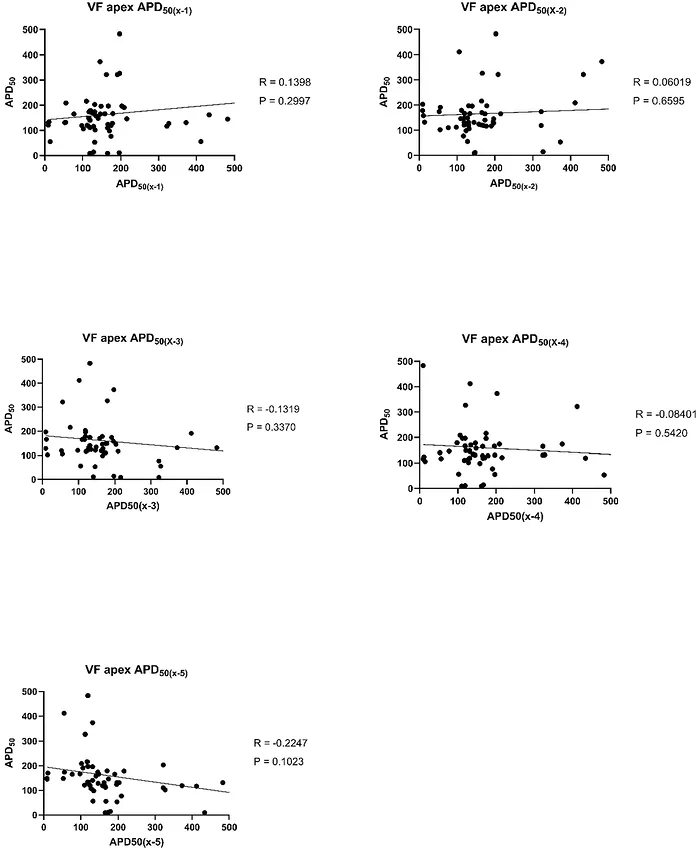
Representative plots showing the relationship between action potential duration (APD) 50 and previous (up to *x* − 5) APD for the first 60 beats following VF initiation as recorded from the apical monophasic action potential (MAP) electrode from an experiment conducted on the 27/2/22. VF scatter plots from calculated APD_50_ (derived from the last normal action potential prior to VF initiation) and preceding APD_50_ (up to *x* − 5) calculated from the *T*_act50_ and *T*_50_. Both the *R*‐ and *P*‐value are shown as calculated by a simple linear regression using GraphPad Prism.

**FIGURE A13:**
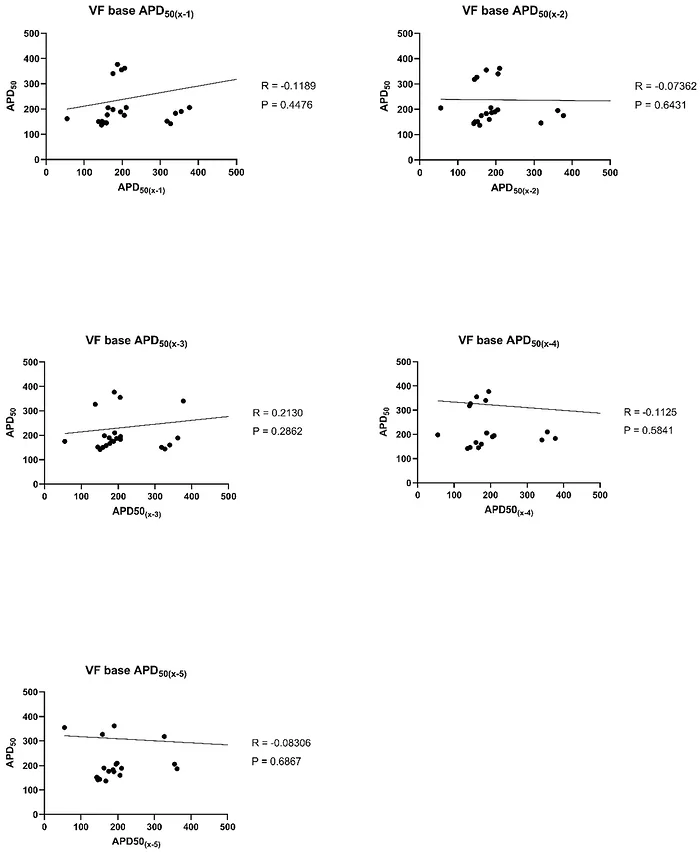
Representative plots showing the relationship between action potential duration (APD) 50 and previous (up to *x* − 5) APD for the first 60 beats following VF initiation as recorded from the basal monophasic action potential (MAP) electrode from an experiment conducted on the 8/3/22. VF scatter plots from calculated APD_50_ (derived from the last normal action potential prior to VF initiation) and preceding APD_50_ (up to *x* − 5) calculated from the *T*_act50_ and *T*_50_. Both the *R*‐ and *P*‐value are shown as calculated by a simple linear regression using GraphPad Prism.

**FIGURE A14:**
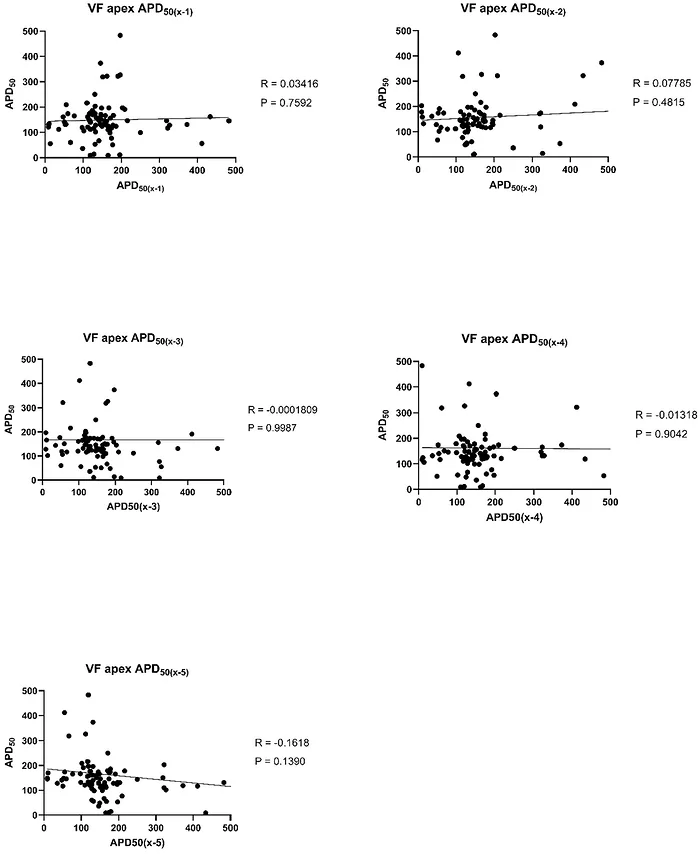
Representative plots showing the relationship between action potential duration (APD) 50 and previous (up to *x* − 5) APD for the first 90 beats following VF initiation as recorded from the apical monophasic action potential (MAP) electrode from an experiment conducted on the 27/2/22. VF scatter plots from calculated APD_50_ (derived from the last normal action potential prior to VF initiation) and preceding APD_50_ (up to *x* − 5) calculated from the *T*_act50_ and *T*_50_. Both the *R*‐ and *P*‐value are shown as calculated by a simple linear regression using GraphPad Prism.

**FIGURE A15:**
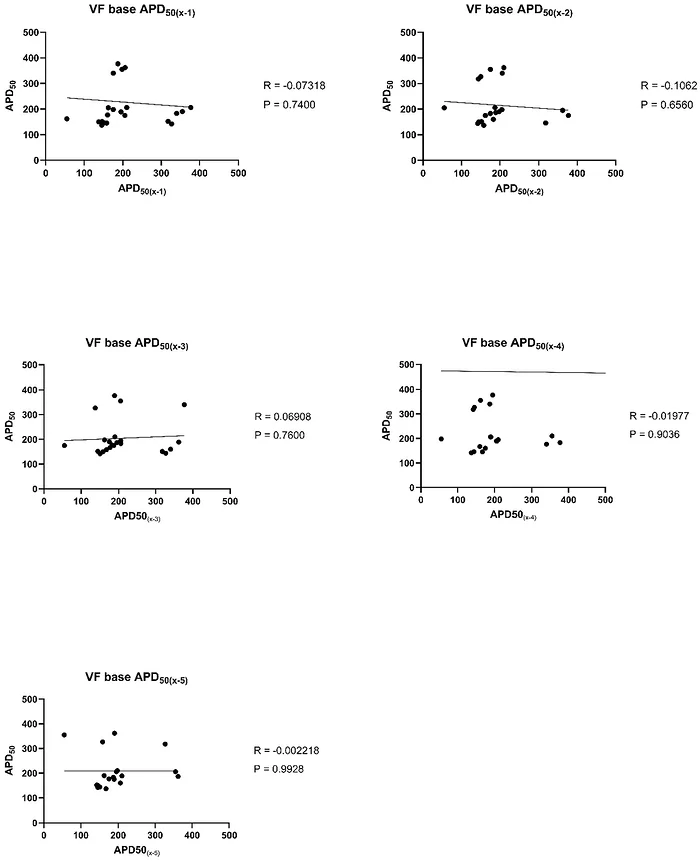
Representative plots showing the relationship between action potential duration (APD) 50 and previous (up to *x* − 5) APD for the first 90 beats following VF initiation as recorded from the basal monophasic action potential (MAP) electrode from an experiment conducted on the 8/3/22. VF scatter plots from calculated APD_50_ (derived from the last normal action potential prior to VF initiation) and preceding APD_50_ (up to *x* − 5) calculated from the *T*_act50_ and *T*_50_. Both the *R*‐ and *P*‐value are shown as calculated by a simple linear regression using GraphPad Prism.

## Data Availability

All data that support the findings from this study can be made available from the authors upon reasonable request. No generative AI (GAI) tool has been used for the preparation of this manuscript.
